# Prior-informed population pharmacokinetic-pharmacodynamic modeling of dexamethasone in horses

**DOI:** 10.1007/s10928-026-10062-7

**Published:** 2026-09-26

**Authors:** Ruihong Yu, Pierre-Louis Toutain, Carl Ekstrand, William J. Jusko

**Affiliations:** 1 Division of Pharmacokinetics, Pharmacodynamics, and Systems Pharmacology, Department of Pharmaceutical Sciences, School of Pharmacy and Pharmaceutical Sciences, State University of New York at Buffalo, 160 Hayes Road, 404 Pharmacy Building, Buffalo, NY 14214-8033 USA; 2 Department of Comparative Biomedical Sciences, The Royal Veterinary College, University of London, London, UK; 3 Department of Animal Biosciences, Swedish University of Agricultural Sciences, Uppsala, Sweden

**Keywords:** Dexamethasone, Sex difference, Informative priors, Bayesian estimation, Horse

## Abstract

Dexamethasone (DEX) is widely used in equine practice with diverse studies having characterized its pharmacokinetics (PK) and pharmacodynamics (PD) including our published comprehensive meta-analysis. Individual DEX concentrations in plasma and urine and serum cortisol from five studies (*n* = 32 horses) were analyzed using a minimal physiologically-based PK (mPBPK) model and a circadian baseline-indirect response PD model. The previous meta-analysis provided complex structural models to assess sources of variability across an array of studies involving intravenous (IV), intramuscular (IM) and intra-articular (IA) dosing of various formulations. Here we compare three population estimation approaches: first-order conditional estimation with interaction (FOCEI), FOCEI with informative priors (FOCEI-Priors), and full Bayesian estimation. The primary covariate found was hepatic clearance of DEX being 17% higher in adult female horses (0.59 L/h/kg) than in geldings (0.49 L/h/kg). Between-subject variability was identified for some parameters. Standard FOCEI required extensive fixing of parameters to achieve convergence. FOCEI-Priors exhibited robust estimation performance, resulting in well-captured DEX PK and cortisol profiles, precise parameter estimates, and satisfactory model diagnostics. Bayesian estimation failed in the posterior exploration for cortisol PD, likely due to its greater sensitivity to prior specification and data variability. This work demonstrated effective population extensions of complex, prior-informed, mechanistic mPBPK/PD models to confirm determinants and assess variability of DEX disposition and adrenal effects in horses.

**Supplementary Information:**

The online version contains supplementary material available at https://doi.org/10.1007/s10928-026-10062-7.

## Introduction

Dexamethasone (DEX) is a widely used synthetic glucocorticoid with potent anti-inflammatory and immunosuppressive properties, making it an essential therapeutic agent in both human and veterinary medicine. In horses, DEX is commonly administered to treat airway inflammation, musculoskeletal disorders, and other inflammatory conditions. Use of DEX presents unique challenges in racehorses as corticosteroid treatments are subject to strict regulatory oversight due to potential effects on animal performance and welfare. Population pharmacokinetic (popPK) and pharmacodynamic (popPD) modeling has recently been advocated to optimize dosing strategies and estimate withdrawal times before treated horses are allowed to race [1].

The PK/PD of any drug can be inherently variable across individual horses due to differences in demographic, physiological, and management-related factors. For example, acetaminophen clearance was greater in mares than stallions [2]. Seasonal variations in basal adrenocorticotropic hormone (ACTH) concentrations occur in healthy horses and ponies, along with additional DEX modulation of cortisol suppression related to age and sex [3]. Typical equine pharmacology studies encounter diverse experimental and field conditions with variable dosing regimens, formulations, sampling schedules, and study objectives. Multiple pharmaceutical forms of DEX are available including the free alcohol and ester derivatives such as DEX sodium phosphate (DEX_PHO) and DEX isonicotinate (DEX_ISO). These may be administered to horses by intravenous (IV), oral (PO), intra-articular (IA), intramuscular (IM), dermal (as an ointment) [4], topical [5] and nebulization [6] routes. Previous studies with DEX often had limited objectives and multiple sources without cross-study comparisons and formal population assessments.

Population modeling using a PBPK/PD structure has been successfully performed for horses, but identification of covariate and random effects encountered a large number of parameters and limited information content in the small equine dataset [7].

The popPK/PD modeling of complex mechanistic systems can be daunting. Conventional methods frequently encounter parameter identifiability issues, numerical instability, and need for extensive parameter fixing, thereby limiting the ability to quantify covariate effects. A key challenge lies in how prior information can be derived and transferred in a consistent and scientifically justified manner across different stages of model development. Our mPBPK/PD models of DEX in horses using a naïve-pooled meta-analysis of aggregated data [7] worked well for various dosing routes and forms. Data for individual horses from part of these studies became available to assess popPK/PD methodology and seek sources of variability. Our goal was to implement our comprehensive mechanistic mPBPK/PD structure into a nonlinear mixed-effects framework. We compared three population estimation approaches: first-order conditional estimation with interaction (FOCEI), FOCEI with informative priors (FOCEI-Priors), and fully Bayesian estimation. Although Bayesian methods provide a natural framework for incorporating prior information, the FOCEI approaches offer a pragmatic compromise by combining the robustness of classical estimation methods with the stabilizing influence of prior knowledge. This work therefore explores these methodologies for seeking estimations in complex population analyses as well as delineates sources of variability in DEX PK/PD.

## Methods

### Data sources

The popPK/PD analysis integrated raw data from five independent studies of DEX conducted in horses (Table 1), which were originally published with varied purposes [8–12]. These studies encompassed diverse DEX dosing regimens, enabling characterization of DEX PK and PD across several exposure conditions. The combined population exhibited some heterogeneity in animal demographics, which allowed systematic exploration of covariate effects on both PK and PD parameters. The datasets included three data types: DEX concentrations in plasma, DEX concentrations in urine, and cortisol concentrations in plasma as a PD biomarker. The DEX and/or cortisol in plasma and urine were analyzed and quantified by using either ultra high performance liquid chromatography–tandem mass spectrometry (UHPLC–MS/MS) or HPLC. All concentrations used were above the study-specific lower limit of quantitation (LLOQ).

**Table 1:** Summary of data sources

Study ID-Year	Study Design	Breed	Age, yr	No.Sex	BW (kg)	Dosing Route	Dosage, Form	Data Type
1-1981	Randomized, cross-over	Saddlebred	7 ~ 15	1 F/5 G	484 ~ 700	IV	0.05 mg/kg DEX	DEX and CTS in plasma (Start at 7 ~ 9 AM), DEX in urine
							0.05 mg/kg DEX_ISO	
2-2010	Single-dose, parallel	Standardbred	3 ~ 16	6 G	430 ~ 545	IM	0.0237 mg/kg DEX_ISO	DEX, CTS in plasma (Start at 9 AM), DEX in urine, CTS baseline
3-2012	Randomized, multi-period, cross-over	Standardbred	6 ~ 20	4 F/2 G	430 ~ 584	IV	0.1 µg/kg + 0.0233 µg/kg/h*3 h DEX_PHO	DEX, CTS in plasma (Start at 9:30 ~ 10:30 AM), CTS baseline
							1 µg/kg + 0.233 µg/kg/h*3 h	
							10 µg/kg + 2.33 µg/kg/h*3 h	
4-2007	Randomized, cross-over	Saddlebred	5 ~ 17	2 F/6 G	430 ~ 618	IV	0.008 mg/kg DEX	DEX in plasma and urine
5-2013	Randomized, placebo-controlled, cross-over	Standardbred	3 ~ 9	3 F/3 G	431 ~ 538	IA	0.01 mg DEX_PHO	DEX and CTS in plasma (Start at 11:30 AM), CTS baseline
							0.1 mg DEX_PHO	
							1 mg DEX_PHO	
							0.03 mg DEX_PHO	DEX and CTS in plasma (Start at 11 AM), CTS baseline
							0.3 mg DEX_PHO	
							3 mg DEX_PHO	

IV, intravenous; IM, intramuscular; IA, intra-articular; DEX_ISO, DEX isonicotinate; DEX_PHO, DEX sodium phosphate; CTS, cortisol. Sex: F = Mare (Female), G = gelding

All dosages are DEX-equivalents

### Structure model

**Fig. 1:**
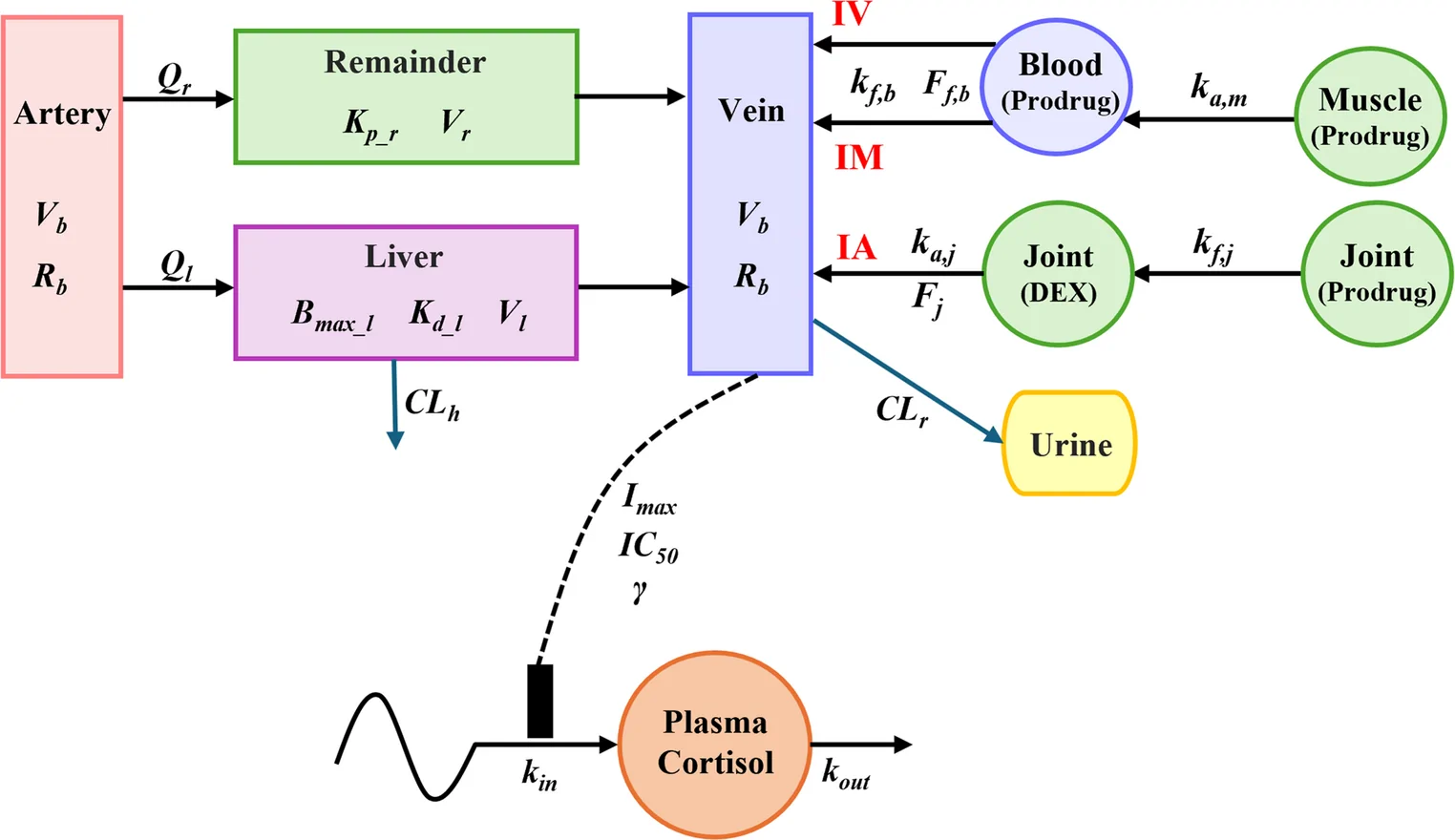
Structural model of dexamethasone (DEX) disposition after intravenous (IV), intramuscular (IM), and intra-articular (IA) dosing and its suppressive effect on circadian cortisol secretion in horses. Symbols are defined in Tables 2 and 3

The structural model (Fig. 1) was described in our previous meta-analysis [13], which was developed using aggregated, mean concentration-time and response–time data digitized from a broad range of published studies and implemented using a naïve pooled approach. The established mPBPK model for DEX consisted of blood, liver and lumped remainder tissue compartments and incorporated first-order prodrug conversion and drug absorption, linear hepatic and renal elimination, and nonlinear hepatic drug binding. The PD model for circadian rhythms and DEX-induced cortisol response was characterized as indirect response model (IDR) I assuming inhibition of adrenal cortisol secretion, with incorporation of a circadian rhythm baseline and a stress-induced early pulse.

**Table 2:** Pharmacokinetic parameters: fixed- and random-effect variabilities for FOCEI-Priors population estimation approaches

Parameters		Unit	Definition	Published	FOCEI-Priors	
					Estimate	RSE% [Shrinkage]
Fixed-effect parameters						
*CL*_*h_gelding*_		L/h	Hepatic clearance in 500-kg geldings and females	236.8	242.8	0.2%
*CL*_*h_female*_		L/h			297	0.2%
*CL*_*r*_		L/h	Renal clearance in 500-kg horses	2.845	3.430	0.7%
*θ*_*BW_CL*_		-	Power exponent of body weight effect on clearances	-	0.75	FIX
*B*_*max_l*_		ng/mL	Drug binding capacity in liver	140.1	121	8.8%
*K*_*d_l*_		ng/mL	Equilibrium dissociation constant in liver	0.269	0.387	8.3%
*K*_*p_r*_		-	Tissue-to-plasma partition coefficient in remainder	1.09	1.04	1.9%
*R*_*b*_		-	Blood-to-plasma ratio	0.69 (FIX)	0.852	1.3%
*k*_*f, b_ISO*_		h^− 1^	The rate constant for DEX conversion from DEX_ISO Bioavailability of DEX_ISO in blood	20.59	22.8	16.8%
*F*_*f, b_ISO*_		%		69.5%	81.2%	10.3%
*k*_*f, b_PHO*_		h^− 1^	The rate constant of DEX conversion from DEX_PHO Bioavailability of DEX_PHO in blood	16.67	16.7	FIX
*F*_*f, b_PHO*_		%		100% (FIX)	100%	FIX
*k*_*f, j_PHO*_		h^− 1^	Prodrug conversion rate constant of DEX_PHO in joints	30 (FIX)	30	FIX
*k*_*a, m_ISO*_		h^− 1^	IM absorption rate constant of DEX_ISO IM absorption rate constant of DEX	0.0105	0.0115	2.2%
*k*_*a, m_DEX*_		h^− 1^		0.131	0.131	19%
*k*_*a, j_DEX*_		h^− 1^	Rate constant for DEX absorption in joints Bioavailability of DEX in joints	5.13	4.94	6.9%
*F*_*j_DEX*_		%		100 (FIX)	100	FIX
Between-subject variability (BSV)						
*ω* ^2^_*CLh*_	-		BSV in *CL*_*h*_		0.094	15.3% [13%]
Residual unexplained variability						
Proportional error for plasma concentration					0.45	2.2% [1.2%]
Proportional error for urine concentration					5.44	8.9% [0.1%]

**Table 3:** Pharmacodynamic parameters: Fixed- and random-effect variabilities for FOCEI-Priors population estimation approaches

Parameters		Unit	Definition	Published	FOCEI-Priors	
					Estimate	RSE% [Shrinkage]
Fixed-effect parameters						
*IC*_*50*_	ng/mL		DEX concentration at 50% maximal effect	0.0383	0.0412	4.7%
*I*_*max*_	-		Maximal inhibitory index	1 (FIX)	1	FIX
*γ*	-		Hill coefficient	1 (FIX)	1.19	5%
*R*_*a*_	ng/mL		Amplitude	18.6	17.6	4.6%
*R*_*m1*_	ng/mL		Mean baseline/mesor for Study 2 (IM doses)	93.4	93	0.5%
*R*_*m2*_	ng/mL		Mean baseline/mesor for other studies	47.9	47	1.7%
*T*_*p*_	h (after 8AM)		Acrophase	0.933	0.992	13.8%
*k*_*out*_	h^− 1^		Loss rate constant of cortisol	0.405	0.246	3.3%
*R*_*max_stress*_	ng/mL		Stress-induced maximal cortisol surge	44.8	24.7	5.7%
*τ*	h		Duration of cortisol surge	0.34 (FIX)	0.34	FIX
Between-subject variability (BSV)						
*ω* ^2^_*Rm*_	-		BSV in *R*_*m*_		0.12	14.4% [5.1%]
Residual unexplained variability						
Additive error					20	0.8% [1.1%]

The prodrug (*i* = DEX_PHO and DEX_ISO) changes in blood following IV dosing were

1 $$\frac{d{A}_{blood\_i}}{dt}={-k}_{f,b\_i}\cdot{A}_{blood\_i}\qquad{A}_{blood\_i}(0)={dose}_{i}$$

For IM doses, the prodrug or parent drug (*i* = DEX and DEX_ISO) changes in the dosing depot and blood were:

2 $$\frac{d{A}_{depot\_i}}{dt}={-\:k}_{a,m\_i}\cdot{A}_{depot\_i}\qquad{A}_{depot\_i}(0)={dose}_{i}$$

3 $$\frac{d{A}_{blood\_i}}{dt}={\:k}_{a,m\_i}\cdot{A}_{depot\_i}-{k}_{f,b\_i}\cdot{A}_{blood\_i}\qquad{A}_{blood\_i}(0)=0$$

For IA doses, the time course of prodrug (*i* =DEX_PHO) and DEX amounts in the dosing depot, the synovial fluid (SF) of joints, were:

4 $$\frac{d{A}_{i,SF}}{dt}={-\:k}_{f,j\_i}\cdot{A}_{i,SF}\qquad{A}_{i,SF}(0)={dose}_{i}$$

5 $$\begin{aligned}&\frac{d{A}_{DEX,\:SF}}{dt}={k}_{f,j\_i}\cdot{A}_{i,SF}\cdot\frac{{MW}_{DEX}}{{MW}_{i}}\\&-{\:k}_{a,j\_DEX}\cdot{A}_{DEX,\:SF}\qquad{A}_{DEX,\:SF}(0)=0\end{aligned}$$

The differential equations for DEX concentrations in blood (*C*_*p*_), liver (*C*_*l*_), and remainder tissues (*C*_*r*_) are:

$$\begin{aligned}&\frac{d{C}_{p}}{dt}\cdot{V}_{b}\cdot{R}_{b}={input}_{IV/IM/IA}+{Q}_{l}\cdot\frac{{C}_{l}}{{K}_{pu\_l}}\\&+{Q}_{r}\cdot\frac{{C}_{r}}{{K}_{p\_r}}-{Q}_{co}\cdot{C}_{p}\cdot{R}_{b}{-CL}_{r}\cdot{C}_{p}\qquad{\:C}_{p}(0)=0\end{aligned}$$

$${input}_{IV/IM}=\:{{F}_{f,b\_i}\cdot\;k}_{f,b\_i}\cdot{A}_{blood\_i}\cdot\:\frac{{MW}_{DEX}}{{MW}_{i}}$$

6 $$\:{input}_{IA}=\:\:{{F}_{j\_DEX}\cdot\:k}_{a,j\_DEX}\cdot{A}_{DEX,\:SF}$$

7 $$\:\frac{d{C}_{l}}{dt}\cdot{V}_{l}={\:Q}_{l}\cdot\left({C}_{p}\cdot{R}_{b}-\frac{{C}_{l}}{{K}_{pu\_l}}\right)-{CL}_{h}\cdot\frac{{C}_{l}}{{K}_{pu\_l}}\qquad{C}_{l}(0)=0$$

8 $$\:\frac{d{C}_{r}}{dt}\cdot{V}_{r}={Q}_{r}\cdot\left({C}_{p}\cdot{R}_{b}-\frac{{C}_{r}}{{K}_{p\_r}}\right)\qquad{C}_{r}(0)=0$$

In above equations, *k*_*f*_ is the formation rate constant of DEX from the prodrugs for the indicated dosing routes, and *k*_*a*_ is the absorption rate constant. *F* is bioavailability due to incomplete prodrug conversion or DEX absorption, *V*_*b*_, *V*_*l*_, and *V*_*r*_ are volumes of the indicated compartments; *Q*_*co*_, *Q*_*l*_, and *Q*_*r*_ are cardiac output, total blood flow to liver, and total blood flow to the remainder tissues. The values of the above physiologic parameters in horses are shown in Table S1 and were scaled according to individual body weight. *R*_*b*_ is the blood-to-plasma ratio in horses. The *CL*_*r*_ and *CL*_*h*_ are the renal clearance and intrinsic hepatic clearance, and *K*_*pu_l*_ and *K*_*p_r*_ are the unbound or total tissue/plasma partition coefficients for liver and remainder tissues.

Liver partitioning was depicted as 1/fraction unbound in liver or [14]:

9 $${K}_{pu\_l}=\:\frac{\sqrt{{\left({C}_{l}-{K}_{d\_l}-\:{B}_{\mathrm{m}\mathrm{a}\mathrm{x}\_l}\right)}^{2}+4\cdot{\:K}_{d\_l}\cdot{C}_{l}}-\left({C}_{l}-{K}_{d\_l}-\:{B}_{\mathrm{m}\mathrm{a}\mathrm{x}\_l}\right)}{2\cdot{\:K}_{d\_l}}$$

in which *B*_*max_l*_, *K*_*d_l*_, and *C*_*l*_ represent binding capacity, equilibrium dissociation constant, and total concentration of DEX in the liver.

The bladder was treated as a well-stirred compartment with a constant effective volume $\:{V}_{bc}$; thus, changes of DEX urine concentration (*C*_*u*_) were driven by renal input and urine outflow, with *CL*_*u*_ denoting the urine flow rate.

10 $$\:\frac{d{C}_{u}}{dt}\cdot{V}_{bc}={{CL}_{r}\cdot\;C}_{p}-{{CL}_{u}\cdot\;C}_{u}$$

Endogenous plasma cortisol (CTS) exhibits a symmetrical circadian pattern and was depicted as a cosine function:

11 $$\:{R}_{b\_CTS}\left(t\right)={R}_{m}+{R}_{a}\cdot\;cos\left(\frac{2\pi\:}{T}\cdot\left(t-{t}_{p}\right)\right)$$

where *R*_*m*_ and *R*_*a*_ are the mean baseline and amplitude, *T* is the biorhythmic period (24 h), and *t*_*p*_ is the peak time or acrophase. In the absence of DEX, plasma CTS is governed by a time-varying synthesis, *k*_*in*_(t), and first-order dissipation, *k*_*out*_:

12 $$\:\frac{d{R}_{b\_CTS}}{dt}={k}_{in}\left(t\right)-{k}_{out}\cdot{R}_{b\_CTS}\left(t\right)$$

Combining Eqs. 11 and 12 yields:

13 $$\begin{aligned}&\:{k}_{in}\left(t\right)=\:{k}_{out}\cdot{R}_{m}+{k}_{out}\cdot{R}_{a}\cdot\:cos\left(\frac{2\pi\:}{T}\cdot\left(t-{T}_{p}\right)\right)\\&-\frac{2\pi\:}{T}\cdot{R}_{a}\cdot\:sin\left(\frac{2\pi\:}{T}\cdot\left(t-{t}_{p}\right)\right)\: \end{aligned}$$

The plasma concentration of DEX (*C*_*p*_) was used as the driving force for adrenal suppression. The mechanism-based effects of DEX on CTS were depicted using indirect response model (IDR) I, which assumes inhibition of the production process [15]:

$$\:\frac{d{R}_{DEX\_CTS}}{dt}=\frac{{R}_{max,\:stress}}{\tau\:}+{k}_{in}\left(t\right)\cdot\left(1-\frac{{I}_{max}\cdot{{C}_{p}}^{\gamma\:}}{{{C}_{p}}^{\gamma\:}+{{IC}_{50}}^{\gamma\:}}\right)-{k}_{out}\cdot{R}_{DEX\_CTS}$$

14 $$\frac{{R}_{max,\:stress}}{\tau\:}=0,\:\mathrm{w}\mathrm{h}\mathrm{e}\mathrm{n}\:t\:\ge\:\:\tau\:$$

where *IC*_*50*_ is the plasma concentration of DEX causing 50% inhibition of CTS production, *I*_*max*_ is its maximal inhibition, *γ* is the Hill coefficient, and *R*_*max, stress*_ is the maximal stress-induced response caused by external disturbances such as animal handling, restraint, and injection pain, which operates for the duration of *τ* after drug dosing.

### Population analysis

The PopPK/PD modeling was performed in NONMEM 7.5.0 (ICON Clinical Research LLC, North Wales, PA) using the ADVAN6 subroutine with TOL = 4, implemented via the Pirana 23.10.2 interface (Certara UK Ltd., Sheffield, UK). Three estimation approaches were explored and compared. Approach 1: standard first-order conditional estimation with interaction (FOCEI). Approach 2: FOCEI with informative priors to perform maximum a posteriori estimation (FOCEI-Priors). Informative priors for fixed-effect parameters were specified based on point estimates and associated uncertainty (CV%) obtained from the previous naïve pooled meta-analysis, and a Wishart distribution with the degrees of freedom set to 4 and 1 was applied to the between-subject variability (BSV) and residual unexplained variability (RUV) matrices. Approach 3: a fully Bayesian analysis using Markov chain Monte Carlo sampling (Bayesian-MCMC), to estimate the full posterior distribution of parameters under the same prior specifications as in Approach 2. Four MCMC chains were run with different random seeds, each consisting of 500 burn-in and 1000 post burn-in iterations. A sequential PK/PD modeling strategy was applied: the popPK estimation was first conducted and individual-specific PK parameters were subsequently fixed for the popPD model estimation. The NONMEM control streams for FOCEI-priors/Bayesian-MCMC implementation are provided in the Supplementary Materials. Covariate selection was conducted using a stepwise covariate modeling procedure. During forward inclusion, covariates were retained based on successful minimization and a decrease in the objective function value (OFV) of at least 3.84 (*p* < 0.05, df = 1), corresponding to statistical significance under a chi-squared distribution. Backward elimination was subsequently performed by removing parameters associated with an increase in OFV of less than 10.83 (*p* < 0.001, df = 1). Categorical covariates were incorporated using category-specific parameters, whereas continuous covariates were incorporated using a power model. The BSV was modeled using an exponential structure assuming log-normal parameter distribution. For example, the covariate effect (*θ*_*BW_CLh*_) of BW, with a mean value (MeanBW) of 500 kg, and the BSV(*η*_*CLh*_) on hepatic clearances (*CL*_*h*_) were described as:

$$\:CLh\:=\:TVCLh\cdot{\left(\frac{BW}{MeanBW}\right)}^{{\theta\:}_{BW\_CLh}}\cdot{e}^{{\eta\:}_{CLh}}$$

Regarding RUV, proportional and combined proportional-additive error models were explored for the PK component, whereas additive and combined error model were explored for the PD component. No data censoring or imputation procedures were conducted.

### Model evaluation and diagnostics

The final model selection within and across approaches were evaluated based on the OFV, standard goodness-of-fit diagnostics, and precision of parameter estimates. Shrinkage values below 30% were considered acceptable. As no external validation dataset was available, internal model evaluation was also conducted using simulation-based predictive checks with 800 replicates. For Bayesian analysis, convergence and sampling adequacy were additionally assessed using trace and density plots, the Gelman–Rubin statistic (^R-hat), and effective sample size (ESS) estimates for both bulk and tail components. All diagnostic plots and Bayesian-related computations were generated by R packages (ggplot2, posterior) using R Studio 2025.09.2 + 418.

## Results

### Descriptive summary of the population datasets

The datasets included in the population analysis are summarized in Table 1, which presents study descriptions and animal demographics. The pooled dataset comprised five studies conducted between 1981 and 2013, encompassing randomized cross-over, parallel, and placebo-controlled designs, with 6 to 8 horses per study, for a total of 32 individual subjects. The cross-study animal cohorts varied in breed (Saddlebred and Standardbred), age (3 to 20 years), sex (10 mares and 22 geldings), and body weight (430 to 700 kg), and these factors were systematically evaluated as potential covariates. DEX was administered as three formulations, including free alcohol, isonicotinate (ISO), and sodium phosphate (PHO), through multiple routes and across a range of dosages: IV bolus and/or infusion (0.17 µg/kg to 0.05 mg/kg), IM injection (0.024 mg/kg), and IA injection (0.01 to 3 mg). A total of 623 DEX plasma and 95 DEX urine concentrations were included in the popPK analysis and 1,063 plasma cortisol concentrations were in the popPD analysis.

### Population PK following FOCEI-priors estimation

**Fig. 2:**
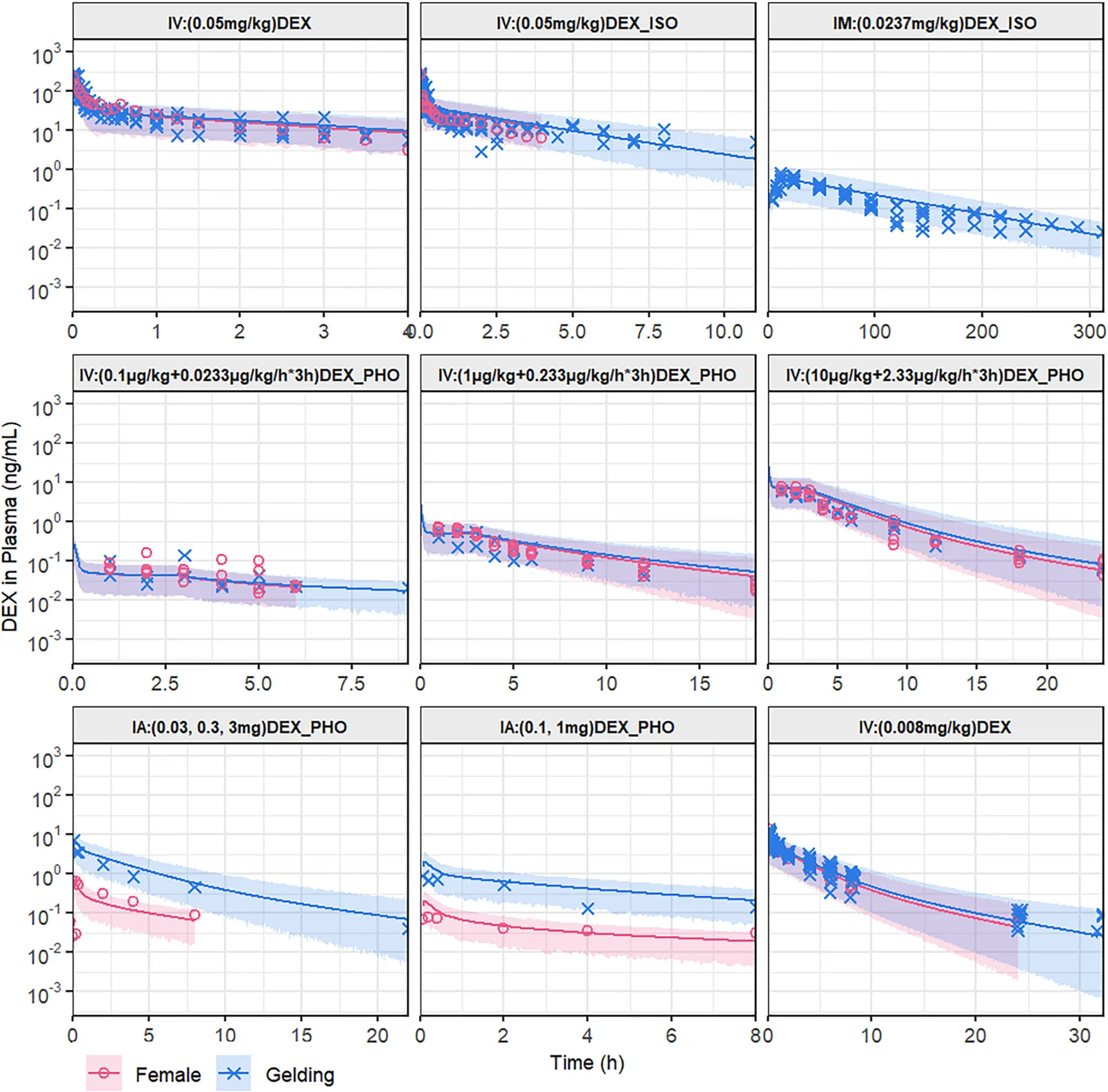
Plasma dexamethasone (DEX) concentration-time profiles. Circles and crosses are observed plasma concentrations, continuous lines and shaded bands depict the median and 5th~95th percentiles of individual predictions from 800 simulations using the FOCEI-Priors model. Data are shown from mares in pink and geldings in blue. Dosing routes, prodrug forms, and equivalent DEX dosages are indicated in the title strip of each panel. IV, intravenous; IM, intramuscular; IA, intra-articular; DEX_ISO, DEX isonicotinate; DEX_PHO, DEX sodium phosphate; DEX, DEX free alcohol

**Fig. 3:**
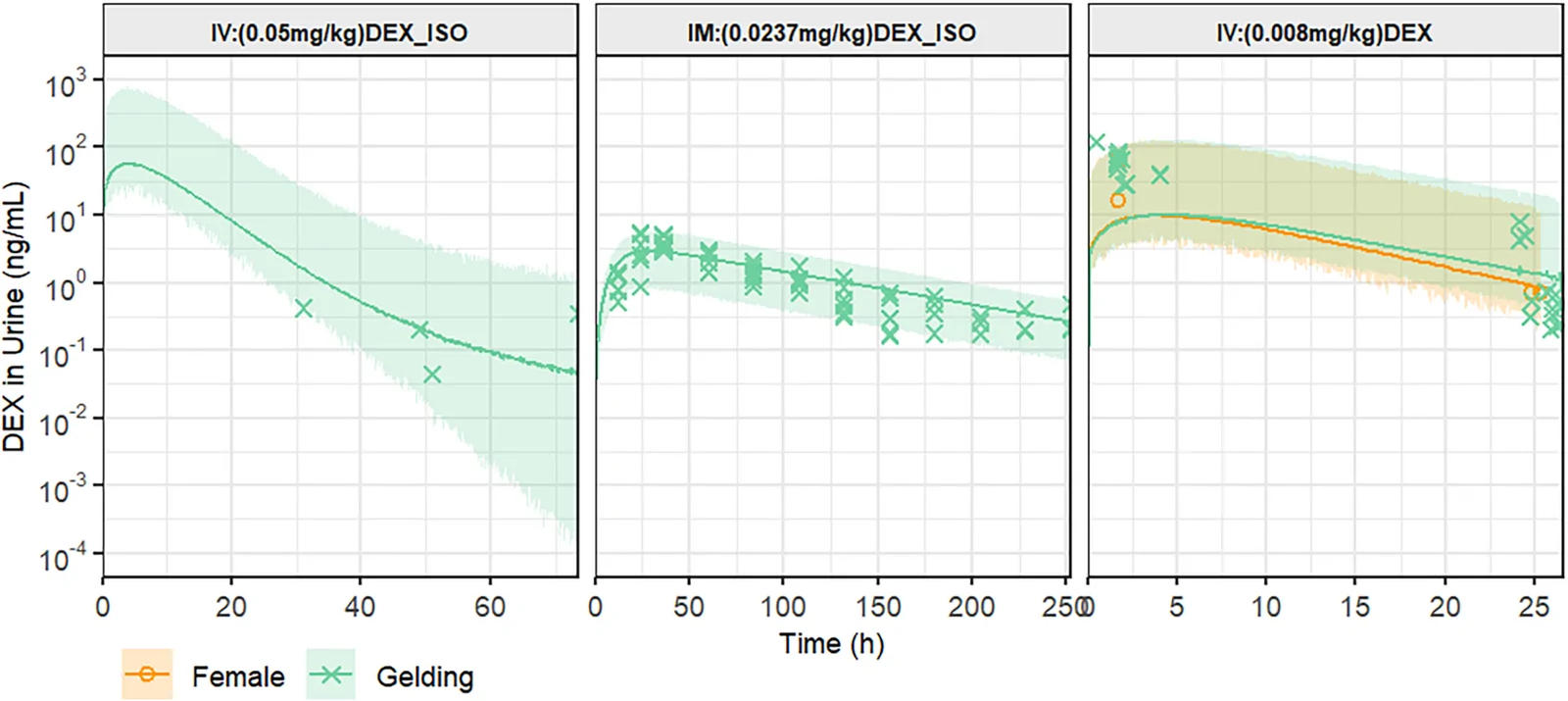
Dexamethasone (DEX) concentration-time profiles in horse urine following the indicated routes and forms of DEX administration. Circles and crosses are observed urine concentrations, continuous lines and shaded bands depict the median and 5th~95th percentiles of individual predictions from 800 simulations using FOCEI-Priors model. Data are shown for mares in orange and geldings in green

The observed and population model-predicted plasma concentration-time profiles of DEX, grouped by administration route and dose, based on FOCEI-Priors estimation, are shown in Fig. 2. The corresponding urine concentration-time profiles are in Fig. 3. The majority of the observed concentrations were well captured by the simulation-based prediction intervals, and the central trends of the DEX exposure profiles were adequately characterized. Other conventional diagnostics for the PK are shown in Fig. S1 (from FOCEI-Priors) and S2 (from Bayesian-MCMC). Conditional weighted residuals were centered at zero and were mostly within the ± 2 range, although slight negative trends were observed as concentrations or time increased, supporting acceptable convergence and estimation performance.

The IV plasma concentration-time profiles (Fig. 2) were polyexponential with a terminal phase extending to 36 h and an apparent half-life of approximately 67 h. Following IM DEX_ISO, measurable DEX concentrations were observed up to 320 h. Plasma sampling after IA dosing was sparse, and concentrations during the terminal phase were frequently BLQ at lower doses. However, measurable concentrations were adequate up to 24 h. Female horses (mares) tended to exhibit lower DEX concentrations compared with geldings. The urine concentration data (Fig. 3) were most robust after IM DEX_ISO dosing and confirmed the prolonged plasma concentrations. Urine data after IV DEX dosing was fragmentary but consistent with the plasma profiles.

The PK parameter estimates from the previous naïve pooled analysis and the current analysis using FOCEI-Priors estimation, together with the random-effect variabilities, are listed in Table 2. The conversion rate constant (*k*_*f, b*_) of DEX_ISO in blood was estimated at 22.8 h^− 1^, with corresponding half-life (*t*_*1/2*_) being less than 2 min, indicating extremely rapid conversion to DEX in blood. The estimated *F*_*f, b_ ISO*_ of 81.2% is close to our previous 69.5% and suggests a 19.8% systemic loss prior to activation. The IM absorption rate constants (*k*_*a, m*_) of DEX in ISO and free alcohol forms were 0.0115 and 0.131 h^− 1^ (*t*_*1/2*_ = 57 and 5.3 h), which are consistent with those from previous analysis (0.0105 and 0.131 h^− 1^). These findings indicate a markedly slower IM absorption of DEX-ISO, as previously found, and are reflected in the prolonged terminal phase driven by flip-flop kinetics (Fig. 2, Panel 3). The absorption of DEX from joints was rapid, as indicated by the *k*_*a, j*_ of 4.94 h^− 1^ here and 5.13 h^− 1^ previously. The bioavailability of DEX_ISO in blood (*F*_*f, b_ISO*_), and that of DEX in joints (*F*_*j_DEX*_) were fixed to 100%, and *k*_*f*_ of DEX_PHO in blood and joint was fixed to 16.7 and 30 h^− 1^, as in our previous analysis. This was needed due to either parameter unidentifiability or limited data availability in specific phases or specimens. For the nonlinear DEX binding in liver, *B*_*max_l*_ was 121 ng/mL here and 140 ng/mL previously while *K*_*d_l*_ was 0.39 versus 0.27 ng/mL. These parameters serve as key determinants of the long-lasting terminal phase observed in the DEX PK profiles. The tissue-to-plasma partition coefficient in the remainder compartment (*K*_*p_r*_) was estimated at 1.0 ~ 1.1 across the two analyses. Additionally, the population analysis enabled estimation of horse-specific blood-to-plasma ratio (*R*_*b*_), which was 0.85 and higher than the previously assumed *R*_*b*_ of 0.69 from rats [16].

**Fig. 4:**
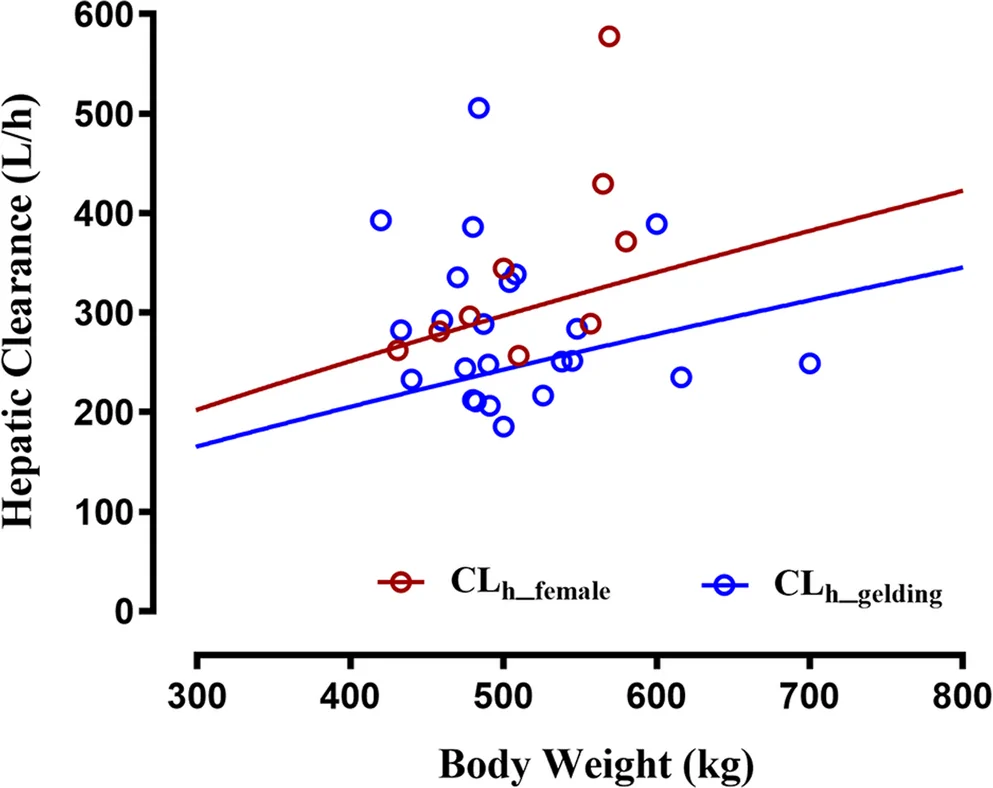
The Body Weight- and Sex-dependent hepatic clearances of dexamethasone in mares and geldings. The lines show the operative popPK relationships with an allometric coefficient set at 0.75

Sex was identified as a significant categorical covariate for hepatic clearance (*CL*_*h*_) of DEX, with a 17% difference. For a 500-kg horse, *CL*_*h*_ was 242.8 L/h for geldings (0.486 L/h/kg) and 297 L/h for mares (0.594 L/h/kg). Both values were slightly higher than the estimate of 237 L/h from the naïve pooled analysis for horses with BW ranging from 385 to 700 kg (mean 550 kg), including mares, stallions, and geldings. Renal clearances (*CL*_*r*_) of DEX using FOCEI-Priors was estimated as 3.43 L/h (6.47 mL/h/kg), which is lower than the predicted glomerular filtration clearance (*CL*_*GF*_) of 18.4 mL/h/kg, as the product of unbound fraction (0.175) [17] and equine glomerular filtration rate (GFR, 105 mL/h/kg) [18]. This suggests that DEX undergoes GFR and is subject to passive tubular reabsorption. The effects of BW with power models for *CL*_*h_female*_, *CL*_*h_gelding*_, and *CL*_*r*_ were explored, yielding a shared exponent that repeatedly converged to 0.75, but with poor precision. Consequently, it was fixed to 0.75, a frequent allometric factor for drug clearances within and across species. Inclusion of BW and sex effects on clearance substantially improved model performance with individual fitted values shown in Fig. 4. The *CL*_*h*_ increases with BW with males generally exhibiting higher *CL*_*h*_ than geldings for the very limited BW range in these adult horses.

Relative standard errors (RSE) for all fixed-effect parameter estimates using FOCEI-Priors estimation were below 20%. Because the available PK data supported estimation of only a single BSV term, BSV was evaluated sequentially on the major PK parameters. A shared BSV on hepatic clearance (*η*_*CLh*_) was ultimately retained based on model performance, physiological interpretability, and standard population PK modeling practice, with an estimated variance of 0.094, good precision (RSE = 15.3%) and low shrinkage (13%). Thus sufficient individual-level information was available to support reliable estimation. A proportional residual error model was ultimately selected for the PK component, with estimated variability of 45% for plasma concentrations and 544% for urine concentrations, both with acceptable RSE and shrinkage. The variability in the sparse urine data was notably high, as reflected by the wide prediction intervals shown in Fig. 3. A preferable metric for urine would be excretion rates that obviate the variability in voided urine volumes.

### Comparison of PK estimation approaches

The model development, including variability and covariate selection, was primarily performed using both standard FOCEI and FOCEI-Priors in a complementary manner. Bayesian MCMC estimation was subsequently implemented following the same structure. A comparison of the fixed-effect PK parameter estimates and random-effect variabilities across the three estimation approaches is presented in Table S2. In the absence of prior information and limited data information, standard FOCEI required fixing several parameters to achieve full convergence, leaving only a limited number of parameters identifiable for estimation. Accordingly, estimation was prioritized for the most critical PK parameters, including sex-specific *CL*_*h*_, *CL*_*r*_, and horse-specific *R*_*b*_. However, FOCEI-Priors and Bayesian-MCMC approaches converged reliably and enabled estimation of all parameters. The corresponding objective function values of the three approaches were 1198, 1110, and 1035–1036 (across four chains), indicating increasingly better model fits. The Bayesian-MCMC estimation showed improved diagnostic performance (Fig. S2) compared with FOCEI-Priors (Fig. S1), particularly in the individual prediction versus observation plots. No visually appreciable differences were observed in the fitting plots among the three approaches, therefore, only simulation-based visual checks for the FOCEI-Priors approach are presented (Figs. 2 and 3). In addition, the two prior-informed approaches provided improved parameter precision, reflected by smaller RSE% values compared to standard FOCEI, and yielded parameter estimates more comparable to those from the previous naïve pooled analysis.

FOCEI-Priors and Bayesian MCMC estimations yielded comparable PK parameter estimates and precision, indicating similar model robustness. Bayesian-specific diagnostics shown in Fig. S2 and parameters shown in Table S2 indicated generally acceptable convergence and sampling performance. Trace plots for all fixed-effect and variability parameters (Fig. S3) exhibited good mixing and stable sampling behavior across the four MCMC chains, with no apparent drifts over iterations. Posterior density distributions (Fig. S4) were generally unimodal and largely overlapped across chains. The improved OFV and diagnostic performance of the Bayesian-MCMC approach were significant, primarily driven by a wide BSV for *CL*_*h*_ (*ω*^2^ = 0.13, CV = 37.3%), which was accompanied by a relatively higher *η*-shrinkage (22.5%). This represented the most notable difference compared with the FOCEI-Priors, which yielded a smaller BSV (*ω*^2^ = 0.094, CV = 31.4%) but lower *η*-shrinkage (13%). Most parameter estimates from Bayesian-MCMC exhibited *R̂* values of 1.01, while those for *CL*_*r*_, *B*_*max_l*_, and *K*_*d_l*_ were slightly higher (1.02 ~ 1.03), indicating less favorable convergence diagnostics (Table S2). Tail effective sample size (ESS) estimates measuring efficiency of MCMC were generally ≥ 400 for most parameters, meeting the commonly recommended thresholds for MCMC diagnostics. However, most bulk ESS values fell within the range of 200 ~ 400, indicating somewhat lower sampling efficiency. Overall, even though the two Prior-based estimation approaches performed well and produced comparable results, the Bayesian algorithm exhibited slightly suboptimal MCMC diagnostics, particularly with respect to bulk ESS and *R̂*. Thus, the final PK parameter estimates and BSV obtained from the FOCEI-Priors estimation were used as input parameters for subsequent popPD modeling.

### Population PD

**Fig. 5:**
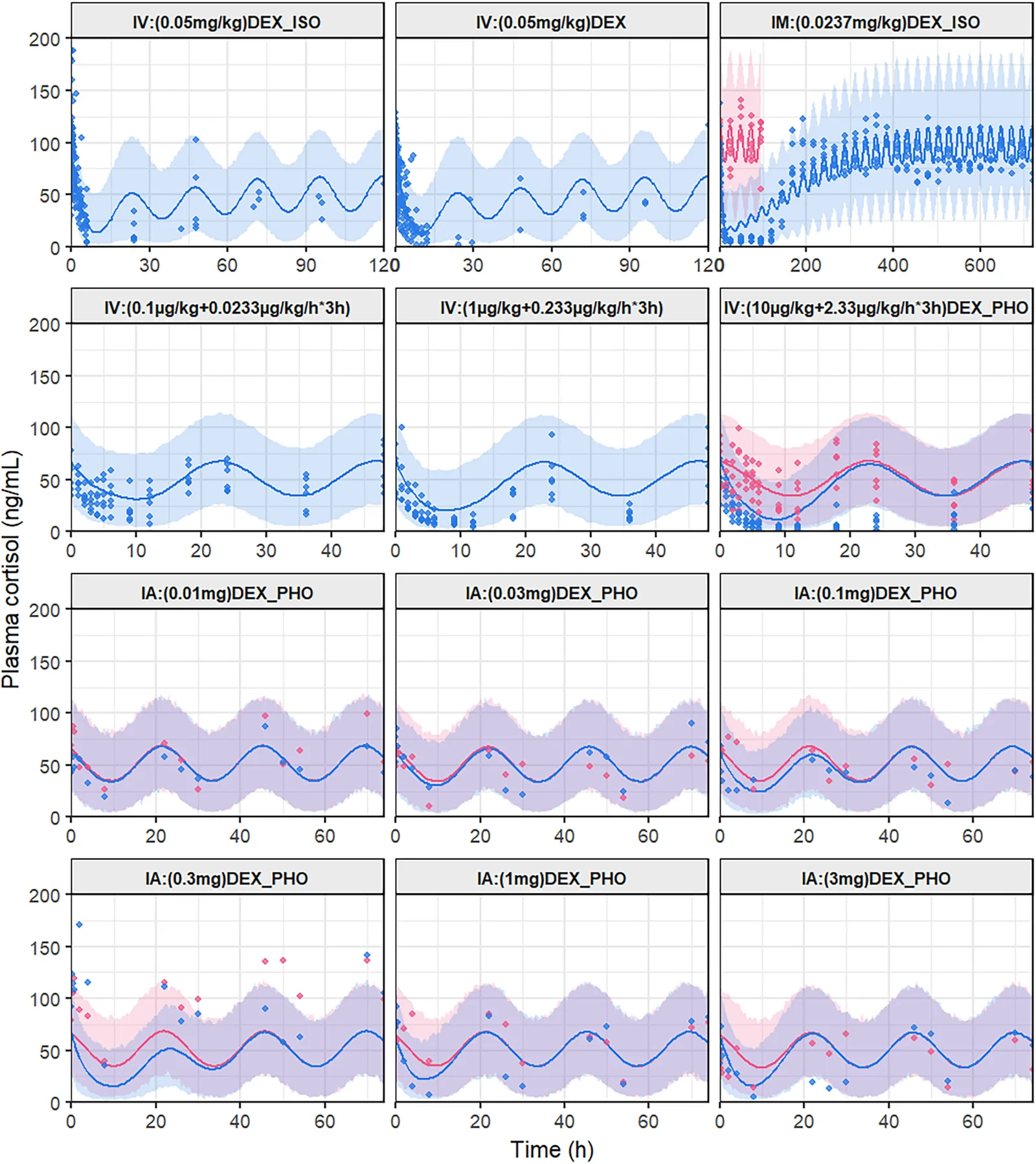
Plasma cortisol concentrations (as response)-time profiles following DEX administration. Symbols are observed cortisol concentrations, continuous lines and shaded bands depict the median and 5th~95th percentiles of individual predictions from 800 simulations using the FOCEI-Priors model. The placebo-baselines are shown in pink and DEX-induced responses in blue

The observed and FOCEI-Priors–based population model–predicted cortisol concentration–time profiles are presented in Fig. 5, with the corresponding diagnostic plots shown in Fig. S5. The placebo panels clearly illustrate the baseline circadian rhythm of cortisol. Larger single doses of DEX produce substantial adrenal suppression, with cortisol concentrations decreasing to near 0 for several hours before returning to the typical circadian rhythm by approximately 36 h. The prolonged PK profiles after IM DEX_ISO dosing produces low cortisol concentrations for more than 100 h, with baseline circadian fluctuations re-emerging after 300 h. As shown in Fig. 5, the triple factors of circadian baseline, DEX inhibition, and the stress-induced surge adequately characterized the general trend of cortisol dynamics. The figure metrics support adequate convergence and reliable estimation performance albeit with a small proportion of unexplained outliers and a wide confidence interval.

The fixed-effect PD parameter estimates and random-effect variability obtained using FOCEI-Priors, together with those from the previous naïve-pooled meta-analysis, are summarized in Table 3. The RSEs for all parameter estimates were below 20%. All fitted parameters were quite similar to those found previously. The endogenous cortisol baseline exhibited a circadian rhythm with a 24-hour periodicity, characterized by a stably estimated amplitude (*R*_*a*_) of 17.6 ng/mL. A categorical, study-dependent effect was identified by both analysis methods for the mean (mesor) cortisol concentration (*R*_*m*_), yielding an estimate of 93 ng/mL in the study with IM dosing and 47 ng/mL in the remaining studies. The acrophase (*T*_*p*_) occurred at 9:00 AM, closely aligning with the observed cortisol peak at 9:11 AM reported in an intensive temporal sampling study involving 10-min intervals [19]. The cortisol loss rate constant (*k*_*out*_) was estimated at 0.25 h^− 1^ (*t*_*1/2*_ of 2.8 h) for these five studies, consistent with the reported range of 0.27 ~ 0.46 h^− 1^ [20]. Assuming complete DEX inhibition of cortisol production (*I*_*max*_ = 1), the *IC*_*50*_ was estimated at 0.041 ng/mL while our previous value was 0.038 ng/mL. The Hill coefficient (*γ*) was 1.12, indicating a slightly steeper concentration-response relationship. Lastly, the typical magnitude of stress-induced response (*R*_*max*_) was 24.7 ng/mL and the duration (*τ*) was assumed to be 20 min.

No significant covariate effect was identified for cortisol response. BSV was included only on *R*_*m*_ in the final model, while the BSV on other response-related parameters, including *IC*_*50*_, failed to converge. The corresponding variance was estimated to be 0.12 (CV = 35.7%), with a good RSE of 14.4% and low shrinkage (5.1%), indicating moderate heterogeneity in individual circadian mesor in horses. The inclusion of residual variability of cortisol data was adequately described by an additive error model, approximately 20 ng/mL. This relatively large additive error may reflect additional sources of variability that were not explicitly accounted for in the model. The most likely explanation is the incursion of random pulse secretion having high amplitudes and short duration (approximately 1 h), on the circadian rhythm, as previously reported [19, 21].

Table S3 compares the fixed-effect PD parameter estimates and random-effect variabilities between standard FOCEI and FOCEI-Priors estimations. Like the population PK analysis, FOCEI-Priors provided greater flexibility in estimating all identifiable PD parameters and improved parameter precision compared with standard FOCEI. The Bayesian MCMC method showed limited convergence performance for the DEX-induced cortisol suppression model under the current settings and prior specification.

## Discussion

### General perspectives

The present work extends our previously published mPBPK/PD model of DEX in horses, originally developed using a naïve pooled meta-analysis, into a nonlinear mixed-effect framework, with a particular focus on evaluating three estimation strategies that leverage available prior information. Parameter estimates and associated uncertainty derived from the earlier meta-analysis were formally incorporated as informative priors to support population analysis of individual-level data collected across five independent studies performed by one research group.

This work enabled a systematic comparison of standard FOCEI, FOCEI-Priors, and fully Bayesian approaches within a unified PK/PD structure, with the aim of assessing the practical benefits and limitations of these different estimation paradigms in handling model complexity, parameter identifiability, and cross-study data heterogeneity. We demonstrated the effective use of a prior-informed population modeling to gain model robustness when transitioning from aggregate to individual-level PK/PD analyses.

### Significant findings through population analysis

Overall, nearly all of the PK and PD parameter estimates obtained from the present population analysis were consistent with, and further confirmed, those previously reported [13], where their rationale and interpretation were discussed in detail. A modest sex-related difference in hepatic clearance of DEX was newly identified with mares (*n* = 9) exhibiting 17% higher clearance than geldings (*n* = 22). Conventional PK studies in humans [22] and camels [23] have reported no apparent sex difference in DEX PK, likely reflecting limited sample sizes. However, methylprednisolone was found to exhibit higher *CL* in women [24]. In contrast, higher *CL* of DEX [16] and methylprednisolone [25] was found in male rats compared to females. In the human liver, DEX and other GCs are extensively metabolized via oxidative pathways mediated by cytochrome P450 (CYP) 3 A enzymes [12, 26]. Sex differences in CYP activity, such as increased activity of human CYP3A in females [27] and male-specific expression of CYP3A2 in rats [28], were posed. Equine CYP3A comprises multiple isoforms that differ from those in humans and rodents [29]. In addition, geldings represent a hormonally distinct group as long-term alterations in endocrine status following castration may influence hepatic metabolism.

### Considerations for PK/PD diagnostics

The PBPK/PD framework required a relatively large number of fixed-effect parameters compared to empirical models, while the diverse prodrug formulations and dosing routes across studies further increased the number of relevant input parameters. Consequently, estimation of substantial BSV was not well supported, as individual predictions showed only modest improvement over population predictions in the diagnostic plots (Fig. S1 and S5). The PK diagnostics showed that deviations from the line of identity were largely attributable to the urine concentration data, consistent with the high residual variability estimated for this endpoint. Owing to time- and individual-dependent hydration status, incomplete collection, and variable voiding intervals, urine sampling in large animal species is intrinsically more variable than plasma sampling. Thus, the observed variability is more plausibly accounted for by residual unexplained variability than by interindividual variability in renal clearance.

For the PD component, deviations in the diagnostic plots were predominantly associated with observations from the IM dose (Fig. 4, Panel 3) and the 0.3 mg IA dose (Fig. 4, Panel 10). During preliminary model testing, inclusion of BSV on the Hill coefficient (*γ*) markedly improved the fit, largely resolving the discrepancy seen with the IM dose, with a decease in OFV of 39. However, this improvement was smaller than achieved in the final model which retained BSV on *R*_*m*_ (∆OFV > 300); the PD dataset was not informative enough to support more than one BSV. The moderate inter-individual variability in *R*_*m*_ is consistent with findings from a human study that reported substantial variability in the mean baseline of cortisol, potentially associated with differences in obesity [30].

### Population estimation strategy

The multiple formulation- and route-specific PK input processes, together with circadian and stress-related components in the PD model, substantially increased model dimensionality. In our previous meta-analysis, model estimation was based on extensive and aggregated mean concentration–time profiles collected across 12 studies and 28 varied doses of DEX. The use of mean data inherently reduced the impact of potentially abnormal observations and attenuated random variability, resulting in cleaner and more stable parameter estimation. In addition, the integration of data from numerous studies spanning a broad dose range substantially increased overall information richness. In contrast, the present analysis relied on individual-level data from 5 studies and 13 varied DEX doses. The successful operation of the high-dimensional mechanistic model critically depended on the use of informative priors derived from our previous meta-analysis. The prior-informed approaches yielded consistent PK parameter estimates, similar uncertainty metrics, and satisfactory goodness-of-fit, indicating that incorporation of informative priors effectively stabilized estimation regardless of the inferential paradigm.

Under standard FOCEI, stable minimization required fixing several parameters, with estimation prioritized for systemic clearance and horse-specific *R*_*b*_. This was expected given the complexity of the model structure and the heterogeneous nature of the pooled dataset. Consequently, the incorporation of informative priors should be viewed not as a refinement, but as a necessary strategy to stabilize estimation and preserve the mechanistic integrity of the model. FOCEI-Priors relies on prior information primarily as a regularization term in point estimation, making it inherently less sensitive to prior width and shape. In contrast, full Bayesian estimation imposes stricter demands on prior specifications as overly diffuse priors can impair sampling efficiency, whereas overly restrictive priors may unduly constrain posterior inferences [31, 32]. A prior sensitivity analysis was conducted by increasing the prior variance four-fold to evaluate the robustness of the model estimates to the strength of the informative priors. The resulting parameter estimates remained generally consistent with those obtained using the original prior specification, indicating that the key model estimates were not strongly driven by the selected prior precision. As expected, widening the priors resulted in moderate increases in the RSE% of most parameter estimates, whereas the overall parameter uncertainty remained within an acceptable range. A modest reduction in the OFV was also observed, and no meaningful improvement was evident in the diagnostic plots. Collectively, these findings support the robustness of the original prior specification. Nevertheless, if the initial prior covariance matrix results in failure of convergence, it may be useful to consider a moderate relaxation of the prior variance as part of the model-development process.

The five studies included in the population model constituted a subset of the datasets used in our meta-analysis, such that the priors represent an internal transfer of information across analytical stages rather than external borrowing. While these priors were sufficiently informative to stabilize both FOCEI-Priors and Bayesian estimation, they appeared overly restrictive for full Bayesian inference for the current PK as reflected by reduced ESSs and less ideal *R̂* for several parameters. Additionally, the posterior exploration of Bayesian estimation is also dependent on the data information content [33]. For the PD, the cortisol response exhibited substantial additive residual variability, with the estimated additive error (20 ng/mL) comparable to, or even exceeding, the amplitude of the circadian rhythm (*R*_*a*_ = 17.6 ng/mL). Under such low signal-to-noise conditions, the reduced information content of individual-level data limited the efficiency of posterior exploration in the Bayesian framework, a limitation that could not be alleviated even by widening prior uncertainty by ± 50% [34] in our prior sensitivity analysis.

### Limitations

This study has several limitations. Both the PK and PD datasets were relatively limited, with small sample sizes and a limited number of subjects, which restricted the ability to estimate multiple BSV terms and to fully explore potential covariate effects. For example, BSV in *T*_*p*_ exists physiologically as a circadian acrophase is well known to vary substantially between individuals even under comparable environmental conditions [35]. The five studies were conducted across seasons (spring, summer, and winter) and a seasonal shifts in the light-dark cycle affecting the circadian rhythm could be expected [3, 36]. However, seasonal differences could not be reliably estimated and was therefore not included in the final popPD model. Although Bayesian estimation was explored, suboptimal MCMC diagnostics limited its use in the final analysis. Additional iterations might have modestly improved Bayesian diagnostics, however, they were not pursued because of the associated computational burden and time requirements. Considering the extent of inherently fluctuating plasma cortisol concentrations and the substantial computational burden, a sequential PK/PD approach was adopted here and previously as is commonly practice rather than simultaneous PK/PD estimation, even though the latter is generally considered more robust and may alter parameter identifiability [37, 38]. Finally, independent datasets were not available for external validation.

## Conclusions

This work provides a practical methodological example of prior-informed population modeling and offers a broadly applicable strategy for the analysis of complex mechanistic PK/PD systems. Informative priors derived from a naïve-pooled meta-analysis were shown to effectively support population PK/PD modeling and vice versa. While FOCEI-Priors and Bayesian approaches yielded comparable PK estimates, FOCEI-Priors offered superior numerical stability and practical feasibility, particularly with the limited data and high residual variability. The PK/PD of DEX from five somewhat diverse studies were well characterized and confirmed our previous assessment, but added insights regarding the role of BW and sex in DEX clearance in adult horses. The resulting population PK/PD framework for DEX may facilitate quantitative dose optimization and support evidence-based therapeutic and regulatory decision-making in clinical and racing equine settings.

## Supplementary Information

Below is the link to the electronic supplementary material.

Supplementary Material 1

## Data Availability

The experimental data used in this report will be available upon reasonable request from Drs. Toutain and Ekstrand.
