# Supplementary material for: Prior-informed population pharmacokinetic-pharmacodynamic modeling of dexamethasone in horses
Source: J Pharmacokinet Pharmacodyn. 2026 Sep 26;53(6):57. doi: 10.1007/s10928-026-10062-7 (PMC13616021; doi:10.1007/s10928-026-10062-7)
Supplement: Supplementary file 1 [file 10928_2026_10062_MOESM1_ESM.pdf]

Supplemental Materials

Prior-Informed Population Pharmacokinetic-Pharmacodynamic Modeling of Dexamethasone in Horses

Ruihong Yu[1], Pierre-Louis Toutain[2], Carl Ekstrand[3], William J. Jusko[1*]

1. Department of Pharmaceutical Sciences, School of Pharmacy and Pharmaceutical Sciences, State University of New York at Buffalo, Buffalo, New York.

2. Department of Comparative Biomedical Sciences, The Royal Veterinary College, University of London, London, United Kingdom.

3. Department of Animal Biosciences, Swedish University of Agricultural Sciences, Uppsala, Sweden

Runing title: Population PK/PD analysis of dexamethasone

*Correspondence to:

William J Jusko, Ph.D., Division of Pharmacokinetics, Pharmacodynamics, and Systems Pharmacology, Department of Pharmaceutical Sciences, School of Pharmacy and Pharmaceutical Sciences, State University of New York at Buffalo, 404 Pharmacy Building, Buffalo, NY 14214-8033. Telephone: 716-645-2855, Fax: 716-829-6569, E-mail: wjjusko@buffalo.edu

Table S1. Physiological parameters utilized in the minimal physiologically based pharmacokinetic model for a representative 550-kg horse.

Parameter	Definition	Value
V_b (L)	Blood volume	38.90 [a]
V_l (L)	Liver volume	7.15 [a]
V_r (L)	The volume of the remainder tissues	504 [a]
V_{bc} (L)	Constant effective bladder volume	4.0 [b]
Q_{co} (L/h)	Cardiac output	1964 [a]
Q_l (L/h)	Blood perfusion to liver	652 [a]
Q_r (L/h)	Blood perfusion to remainder tissues	1312 [a]
CL_{u} (L/h)	Urine flow rate	0.682 [b]

[a] From [1] ; [b] From [2].

Table S2. Pharmacokinetic parameters: Fixed- and random-effect variabilities for three population estimation approaches.

Parameters	Unit	Published	FOCEI		FOCEI-Prior		Fully Bayesian-MCMC				
		Estimate	Estimate	RSE% [Shrinkage]	Estimate	RSE% [Shrinkage]	Estimate	RSE% [Shrinkage]	$\hat{R}$	Bulk ESS	Tail ESS
Fixed-effect parameters											
$CL_{h\_gelding}$	L/h 500 kg	236.8	296	7.3%	242.8	0.2%	242.8	0.2%	1.01	327	505
CL_{h_female}			357	12.9%	297	0.2%	297	0.2%	1.01	293	483
CL_r		2.845	4.08	7.6%	3.430	0.7%	3.429	0.7%	1.02	255	559
BW on CL (exp)	-	-	0.75	FIX	0.75	FIX	0.75	FIX	-	-	-
B_{max_l}	ng/mL	140.1	140.1	FIX	121	8.8%	120	8.9%	1.02	227	487
K_{d_l}	ng/mL	0.269	0.269	FIX	0.387	8.3%	0.384	7.6%	1.03	255	450
K_{p_r}	1	1.09	1.09	FIX	1.04	1.9%	1.06	3.1%	1.01	233	573
R_b	1	0.69 (fix)	0.864	1.5%	0.852	1.3%	0.854	1.3%	1.01	312	601
k_{f,b_ISO}	h^{-1}	20.59	20.6	FIX	22.8	16.8%	23.1	9.2%	1.01	494	729
F_{f,b_ISO}	%	69.5	69.5	FIX	81.2%	10.3%	82.9%	16.1%	1.01	241	313
k_{f,b_PHO}	h^{-1}	16.67	16.7	FIX	16.7	FIX	16.7	FIX	-	-	-
F_{f,b_PHO}	%	100 (fix)	100	FIX	100	FIX	100	FIX	-	-	-
k_{f,j_PHO}	h^{-1}	30 (fix)	30	FIX	30	FIX	30	FIX	-	-	-
k_{a,m_ISO}		0.0105	0.0105	FIX	0.0115	2.2%	0.0115	3.2%	1.01	370	513
k_{a,m_DEX}	h^{-1}	0.131	0.131	FIX	0.131	19%	0.125	21%	1.01	528	477
k_{a,j_DEX}		5.13	5.13	FIX	4.94	6.9%	4.96	8.1%	1.01	568	386
F_{j_DEX}	%	100 (fix)	100	FIX	100	FIX	100	FIX	-	-	-
Between-subject variability											
ω^2_{CLh}		-	0.061	22.3% [16.2%]	0.094	15.3% [13%]	0.13	14.2% [22.5%]	1.00	825	1959
Residual unexplained variability											
Proportional error (plasma)			0.55	1% [0.9%]	0.45	2.2% [1.2%]	0.46	3.3% [4.2%]		-	
Proportional error (urine)			5.15	10.3% [0.1%]	5.44	8.9% [0.1%]	6.07	14.1% [2.6%]		-	
OFV			1198		1110		1035-1036				

Table S3. Pharmacodynamic parameters: Fixed- and random-effect variabilities for two population estimation approaches.

Parameters	Unit	Published estimate	FOCEI		FOCEI-Prior	
			estimate	RSE%[Shrinkage]	estimate	RSE%[Shrinkage]
Fixed-effect parameters						
IC_{50}	ng/mL	0.0383	0.0459	6.4%	0.0412	4.7%
I_{max}	1	1 (fix)	1	FIX	1	FIX
γ	1	1 (fix)	1	FIX	1.19	5%
R_a	ng/mL	18.6	18.6	FIX	17.6	4.6%
R_{m1}	ng/mL	93.42	93.42	FIX	93	0.5%
R_{m2}	ng/mL	47.89	46.8	14.3%	47	1.7%
T_p (after 8 am)	h	0.933	0.933	FIX	0.992	13.8%
k_{out}	h^{-1}	0.405	0.25	7%	0.246	3.3%
R_{max}	ng/mL	44.8	24.5	28.9%	24.7	5.7%
τ	h	0.34 (fix)	0.34	FIX	0.34	FIX
Between-subject variability						
ω^2_{Rm}	-	-	0.117	20.4% [3%]	0.12	14.4% [5.1%]
Residual unexplained variability						
Additive error			20.08	1% [1.1%]	20	0.8% [1.1%]
OFV			7520		7517	

Parameter definitions are provided in Table 3.

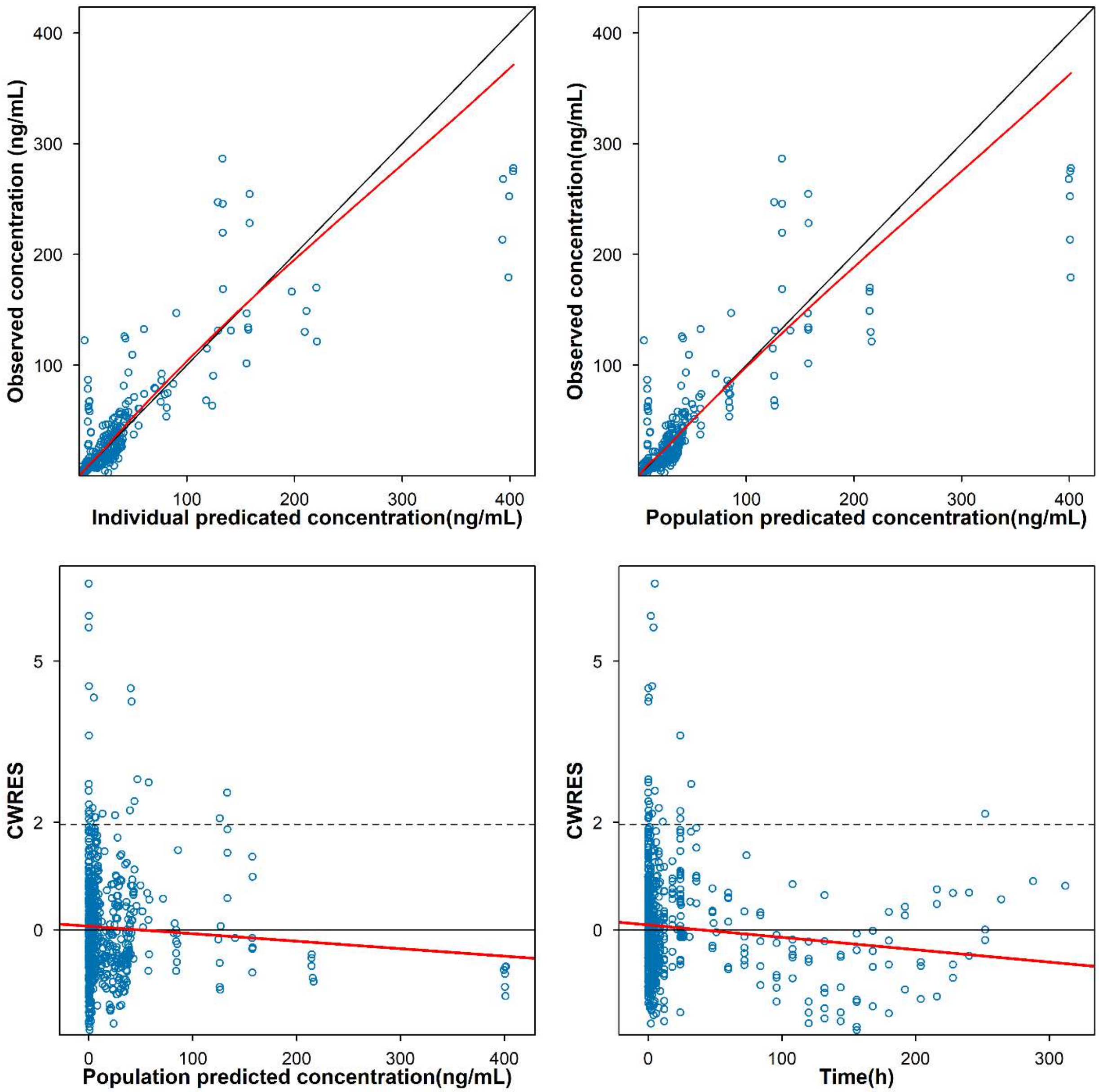

Fig. S1 Diagnostic plots for the dexamethasone pharmacokinetic model in horses for the FOCEI-Priors estimation approach. Red lines are identity lines for upper panels and zero slope lines for lower panels. CWRES, conditional weighted residuals.

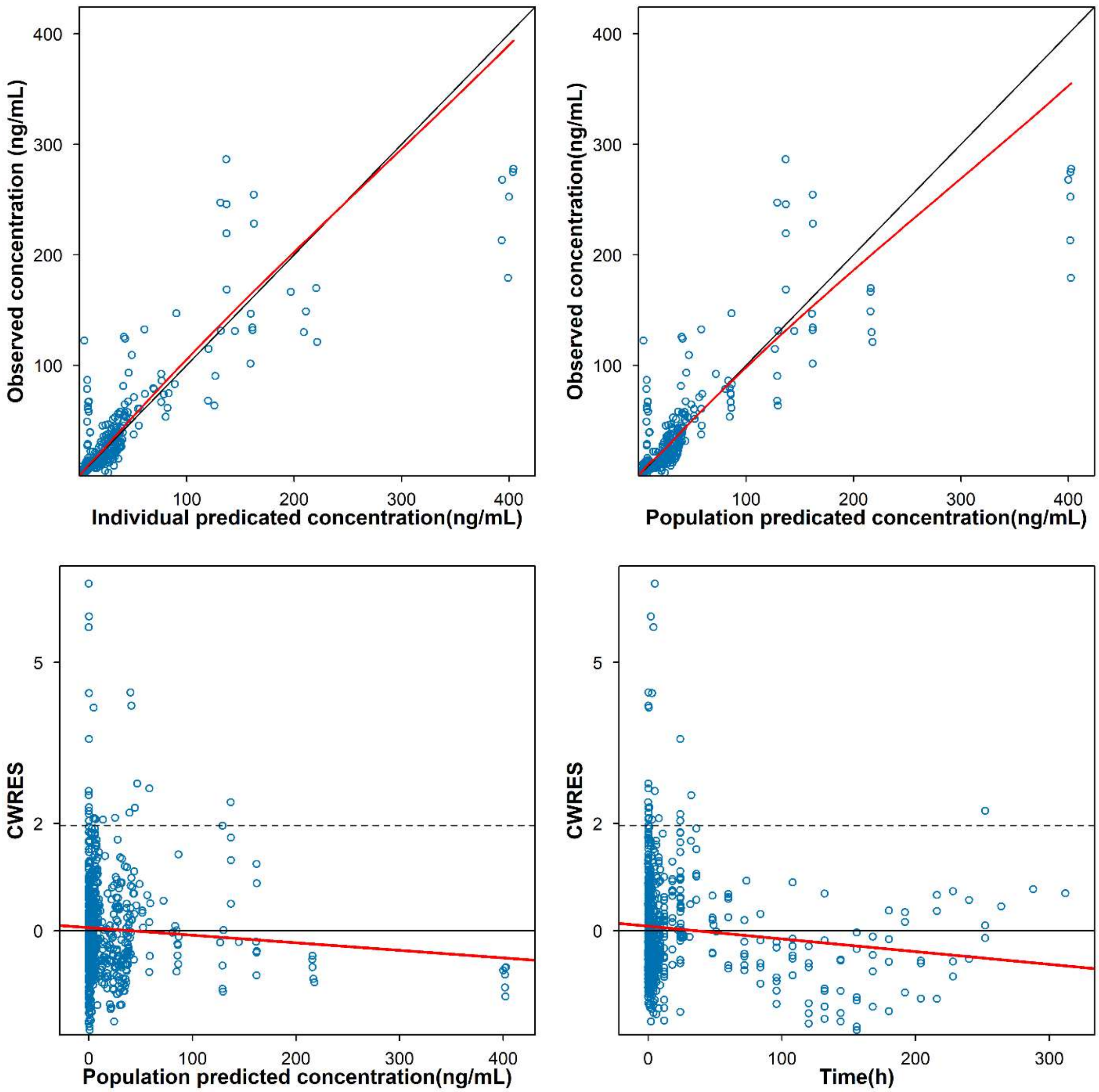

Fig. S2 Diagnostic plots of dexamethasone pharmacokinetic model in horses for the Bayesian-MCMC estimation approach. Red lines are identity lines for upper panels and zero slope lines for lower panels. CWRES, conditional weighted residuals.

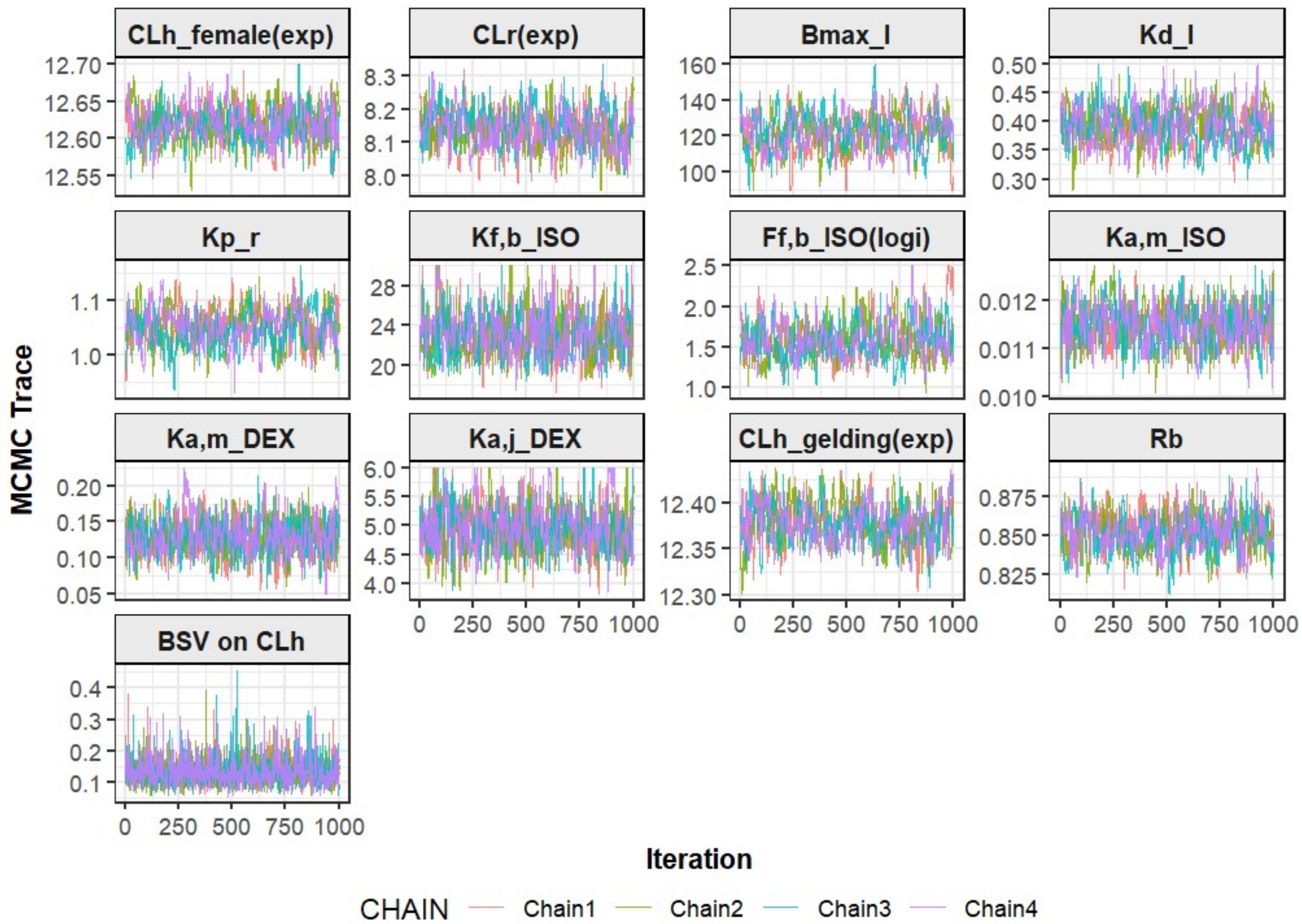

Fig. S3 Trace plots of pharmacokinetic parameters and inter-individual variability from the full Bayesian estimations using Markov Chain Monte Carlo (MCMC) sampling, including 4 chains shown in different colors.

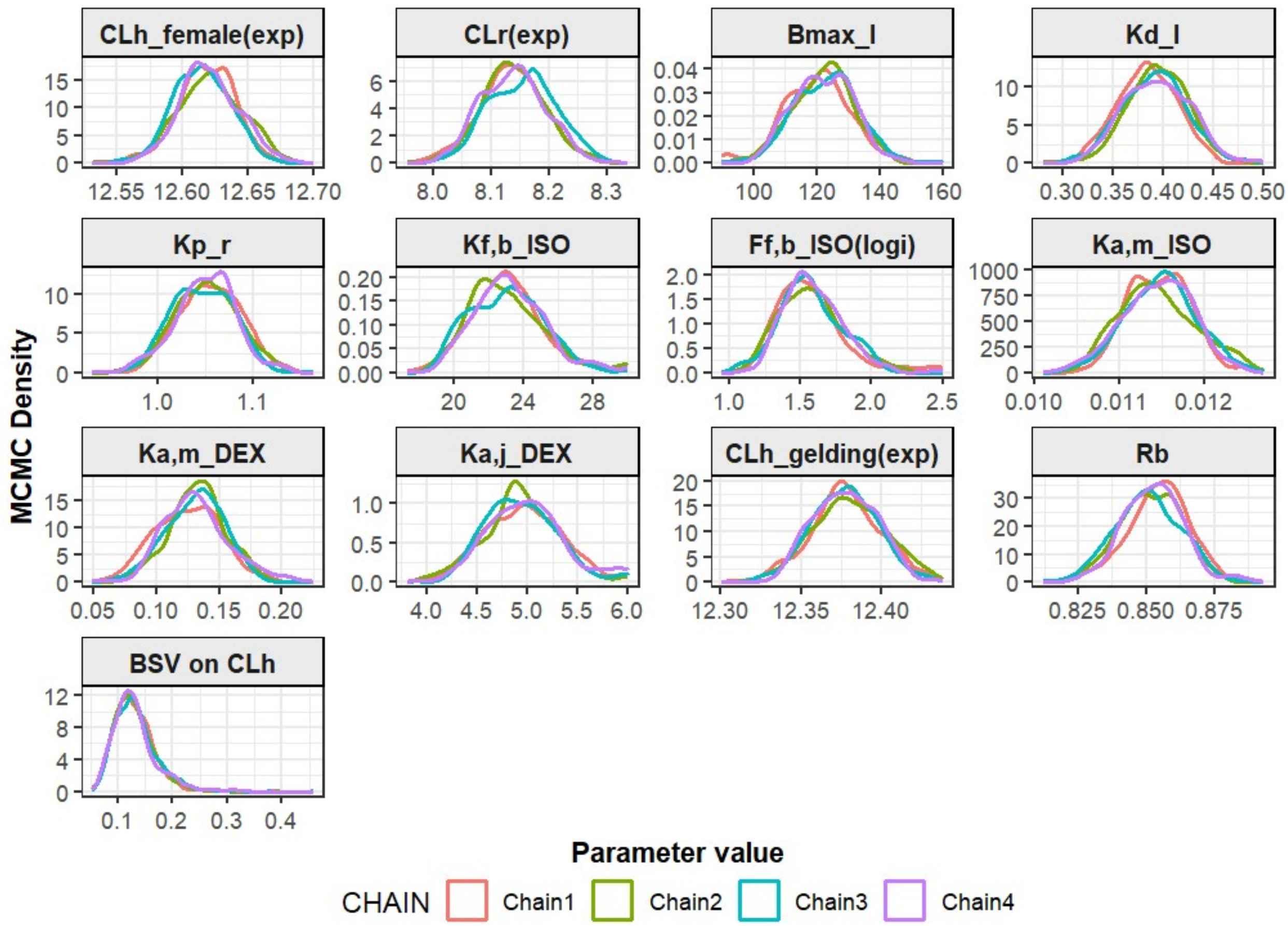

Fig. S4 Density plots of pharmacokinetic parameters and inter-individual variability from the full Bayesian estimations using Markov Chain Monte Carlo (MCMC) sampling.

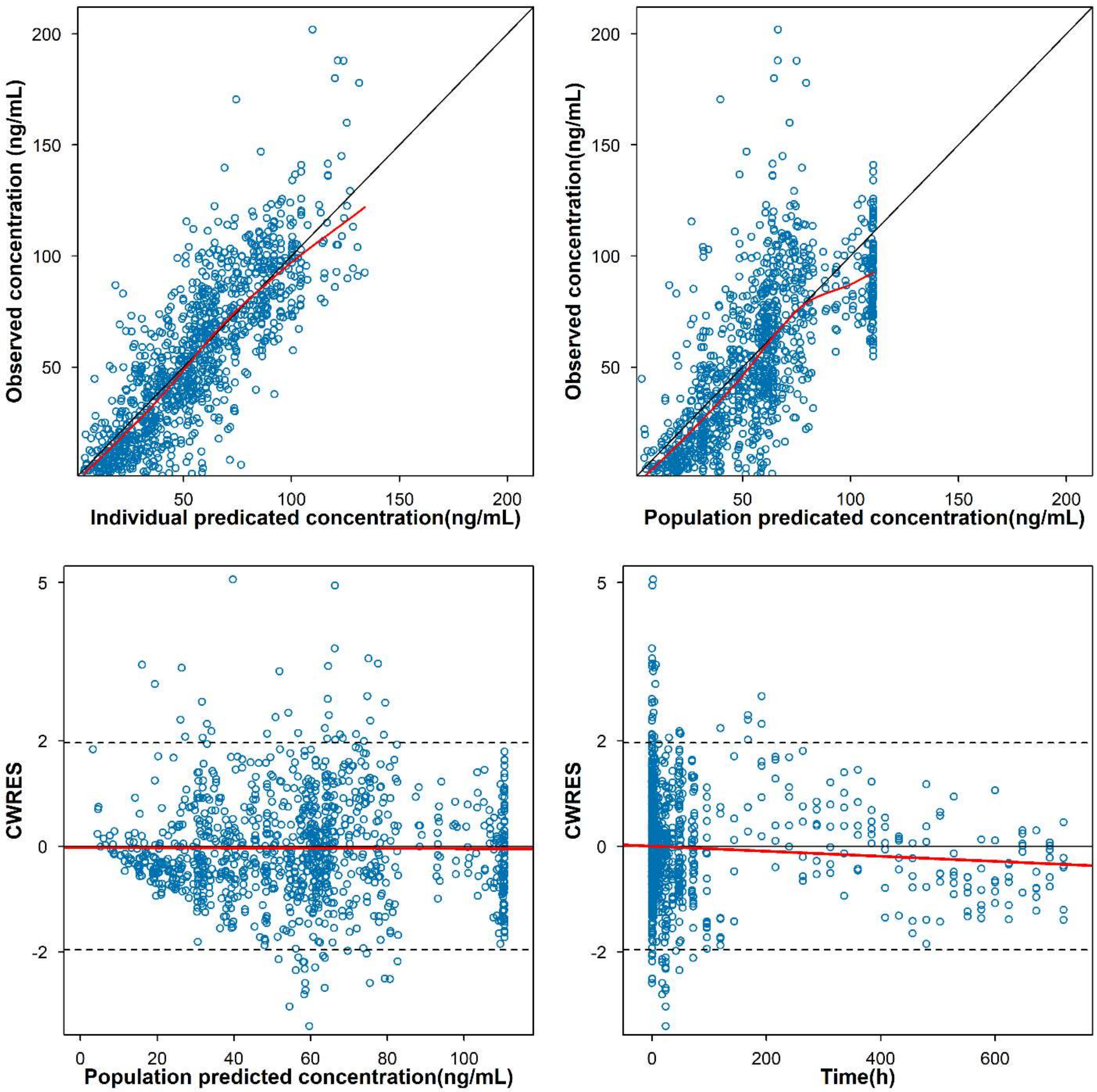

Fig. S5 Diagnostic plots of dexamethasone pharmacodynamic model in horses for the FOCEI-Priors estimation approach. Red lines are identity lines for upper panels and zero slope lines for lower panels.

NONMEM Control Stream of DEX-Pharmacokinetics

```
$PROBLEM DEXPKC3

$INPUT ID PHASE_ID TAD DV TIME AMT RATE CMT DATATYPE MDV EVID CLOCK BW AGE
SEX BREED STUDY_ID ROUTE CONDITION PRODRUG SEASON DATAQUALITY LLOQ DOSE
ERR_TYPE

$DATA DEXPK8.csv IGNORE=#

$SUBROUTINE ADVAN6 TOL=4

$MODEL

COMP = (Prodrug_depot)

COMP = (DEX_depot)

COMP = (DEX_plasma)

COMP = (DEX_liver)

COMP = (DEX_remainder)

COMP = (DEX_urine)

$PK

TVCLhF = exp(THETA(1))     ; CL female (mL/h) at 500 kg

TVCLr =    exp(THETA(2))

TVBmax1 = THETA(3)

TVKd = THETA(4)

TVKp2 = THETA(5)

TVKfs_b = THETA(6)     ; s=DEX_ISO    b=blood

TVKfp_b = 16.67     ; p=DEX_PHO

TVKfp_j = 30     ; j=joint

TVFs_b = 1/(1+exp(-THETA(7)))      ; logit transform

TVFp_b = 1

TVFd_j = 1      ; d=DEX

TVKas_m = THETA(8)

TVKal_m = THETA(9)     ; l=DEX_ALC

TVKad_j = THETA(10)

TVExp_h = 0.75

TVExp_r = 0.75

TVCLhG =    exp(THETA(11))    ;CL Male
```

```
Rb = THETA(12)
;If (SEX.EQ.0) Rb = 0.725               ; Female
;If (SEX.EQ.2) Rb = 0.664               ; Gelding
IF (SEX.EQ.0) THEN
    CLh = TVCLhF * (BW / 500) ** TVExp_h * EXP(ETA(1))
ENDIF
IF (SEX.EQ.2) THEN
    CLh = TVCLhG * (BW / 500) ** TVExp_h * EXP(ETA(1))
ENDIF
CLr = TVCLr * (BW / 500) ** TVExp_r
Bmax1 = TVBmax1
Kd = TVKd* EXP(ETA(1))
Kp2 = TVKp2
If (DOSE.EQ.1) Then    ;    AMT CMT= 2
Kf = TVKfs_b
F_1 = TVFs_b
Ka = 0
End If
If (DOSE.EQ.2) Then ; AMT/Vp/Rb CMT=3
Kf = 0
F_1 = 0
Ka = 0
End If
If (DOSE.EQ.3) Then      ; AMT CMT=1
Kf = TVKfs_b
F_1 = TVFs_b
Ka = TVKas_m
End If
If (DOSE.EQ.4) Then        ; AMT CMT=2
Kf = TVKal_m
```

```
F_1 = 1
Ka = 0
End If
If (DOSE.EQ.5) Then        ; AMT CMT=2
Kf = TVKfp_b
F_1 = TVFp_b
Ka = 0
End If
If (DOSE.EQ.6) Then          ; AMT CMT=1
Kf = TVKfp_j
F_1 = TVFd_j
Ka = TVKad_j
End If
MWd = 392.46
If (PRODRUG.EQ. 0) MWo = 392.46        ; ALC
If (PRODRUG.EQ. 1) MWo = 516.41        ; DEX_PHO
If (PRODRUG.EQ. 2) MWo = 492.54        ; DEX_ISO
Vp = 70.72 * BW          ; mL
V1 = 13.045 * BW
V2 = 916.28*BW
Qco = 3570 * BW          ; mL/h
Q1 = 1184.63*BW
Q2 = 2395.44 * BW
CLu = 1.24 * BW              ; mL/h    (1.24 mL/h/kg)
Vbc = 4000                   ; mL, bladder capacity - trigger micturition
A_0(1) = 0
A_0(2) = 0
A_0(3) = 0
A_0(4) = 0
A_0(5) = 0
A_0(6) = 0
```

```
$DES

Kp1 = (-(A(4) - Kd - Bmax1) + ((A(4) - Kd - Bmax1) ** 2 + 4 * Kd * A(4)) ** 0.5) / (2 * Kd)        ;
nonlinear binding_liver

If (STUDY_ID.EQ.5) Then

DADT(1) = -Kf * A(1)          ; Prodrug Amount_Depot

DADT(2) = Kf * A(1) * MWd / MWo - Ka * A(2)          ; DEX Amount_Depot

DADT(3) = (Ka * F_1 * A(2) + Q1 * A(4) / Kp1 + Q2 * A(5) / Kp2 - Qco * A(3) - CLr * A(3)) / Vp /
Rb    ; DEX conc_blood

Else

DADT(1) = -Ka * A(1)          ; Prodrug Amount_Depot

DADT(2) = Ka * A(1) - Kf * A(2)          ; Prodrug Amount_blood

DADT(3) = (Kf * F_1 * A(2) * MWd / MWo + Q1 * A(4) / Kp1 + Q2 * A(5) / Kp2 - Qco * A(3) - CLr *
A(3)) / Vp / Rb    ; DEX conc_blood

End If

DADT(4) = (Q1 * (A(3) - A(4) / Kp1)) / V1 - (CLh * A(4) / Kp1) / V1             ; DEX conc_liver

DADT(5) = (Q2 * (A(3) - A(5) / Kp2)) / V2                                                  ; DEX
conc_remainder

DADT(6) = (CLr * A(3) - CLu * A(6)) / Vbc                                                   ; DEX conc_urine

$ERROR

  IPRED = F

If (ERR_TYPE.EQ.1) Then

Y = IPRED + IPRED * ERR(1)

Else

Y = IPRED + IPRED * ERR(2)

  ENDIF

  IRES = DV - IPRED

$THETA

(11, 12.6, 14)                              ;[CLhF_exp]        mL/h

(5, 8.14, 9.5)                               ;[CLr_exp] mL/h

(90, 120.1, 160)                                ;[Bmax1]        ng/mL

(0.1, 0.384, 0.5)                              ;[Kd]

(0.8, 1.06, 1.3)                                ;[Kp2]

(5, 23.1, 30)                               ;[Kfs_b]
```

```
(0.5, 1.58, 2.5)                    ;[Fs_b_logit]
(0.0115)                    ;[Kas_m]
(0.125)                     ;[Kal_m]
(1, 4.96, 6)                    ;[Kad_j]
(11, 12.4, 15)                    ;[CLhG_exp]
(0.65, 0.854, 1)                    ;[Rb]
$OMEGA
0.13    ;[CLh]
$SIGMA
25          ;PROP_urine
0.23        ;PRop_plasma
$PRIOR NWPRI
$THETAP         ; fix = informative priors
(12.6 FIX)                  ;[CLhF]
(8.14    FIX)                  ;[CLr]
(140.1    FIX)                 ;[Bmax1]
(0.2693 FIX)                   ;[Kd]
(1.087    FIX)                 ;[Kp2]
(20.59    FIX)                 ;[Kfs_b]
(0.825    FIX)                 ;[Fs_b]
(0.0105 FIX)                   ;[Kas_m]
(0.131    FIX)                 ;[Kal_m]
(5.13      FIX)                ;[Kad_j]
(12.4    FIX)                  ;[CLhG]
(0.853    FIX)                 ;[Rb]
$THETAPV BLOCK(12)
  0.0165 ;    Log(CLhF)
  0.00 0.01 ; Log(CLr)
  0.00 0.00 282.3; Bmax1
  0.00 0.00 0.00 0.00186 ; Kd
  0.00 0.00 0.00 0.00 0.00297; Kp2
```

```
 0.00 0.00 0.00 0.00 0.00 30.8; Kfs_b
 0.00 0.00 0.00 0.00 0.00 0.00 0.834 ; Fs_b
 0.00 0.00 0.00 0.00 0.00 0.00 0.00 0.00000033; Kas_m
 0.00 0.00 0.00 0.00 0.00 0.00 0.00 0.00 0.00062; Kal_m
 0.00 0.00 0.00 0.00 0.00 0.00 0.00 0.00 0.00 0.129; Kad_j
 0.00 0.00 0.00 0.00 0.00 0.00 0.00 0.00 0.00 0.00 0.00531 ; log (CLhG)
 0.00 0.00 0.00 0.00 0.00 0.00 0.00 0.00 0.00 0.00 0.00 0.000143; Rb
$OMEGAP    BLOCK(1)
(0.13)    ;[CLh]
$OMEGAPD    (4.0 FIX)
$SIGMAP
(25 )            ;PROP_urine
(0.23 )        ;PRop_plasma
$SIGMAPD
(1.0 FIX)
(1.0 FIX)
$EST METHOD=COND INTERACTION POSTHOC MAXEVAL=999 NSIG=6 PRINT=5 NOABORT
SIGL=10 MCETA=10        ; FOCEI+Priors

$EST METHOD=BAYES INTERACTION NBURN=500 NITER=1000 ISAMPLE=1 PRINT=500
SEED=32901 CINTERVAL=200 CTYPE=3 SIGDIGITS=4                  ; Bayes

$COV MATRIX=S PRINT=E UNCONDITIONAL

$TABLE ID TIME DV PHASE_ID TAD AMT RATE CMT DATATYPE MDV EVID BW AGE SEX
BREED STUDY_ID ROUTE SEASON DATAQUALITY IPRED PRED ONEHEADER NOPRINT
FORMAT=s1PE13.7 FILE=mytabC3

$TABLE ID TIME DV PHASE_ID TAD CMT DATATYPE MDV PRED IPRED CWRESI CWRES
ONEHEADER NOPRINT FORMAT=s1PE13.7 FILE=sdtabC3

$TABLE ID TIME TAD DV IPRED CMT BW AGE SEX BREED ROUTE CONDITION SEASON CLh
CLr Bmax1 Kd Kp2 Rb ETA1 TVKfs_b TVKfp_b TVFs_b TVKas_m TVKal_m TVKad_j Rb
ONEHEADER NOPRINT NOAPPEND FORMAT=s1PE13.7 FILE=patabC3
```

NONMEM Control Stream of DEX-Pharmacodynamics

```
$PROBLEM DEXPDC8

$INPUT ID PHASE_ID TAD DV TIME AMT RATE CMT DATATYPE MDV EVID CLOCK BW AGE
SEX BREED STUDY_ID ROUTE CONDITION PRODRUG SEASON DATAQUALITY LLOQ DOSE
ETA_CL CLOCK_CCT

$DATA DEXPD6.csv IGNORE=#

$SUBROUTINE ADVAN6 TOL=4

$MODEL

COMP = (Prodrug_depot)

COMP = (DEX_depot)

COMP = (DEX_plasma)

COMP = (DEX_liver)

COMP = (DEX_remainder)

COMP = (DEX_urine)

COMP = (Cortisol)

$PK

TVIC50 = THETA(1)

TVImax = 1

TVGama = THETA(8)

TVRa = THETA(2)

IF (STUDY_ID .EQ. 2) THEN

TVRm = THETA(3)    ;Rm1

ELSE

TVRm = THETA(7)    ;Rm2

ENDIF

TVtp = THETA(4)

TVKout = THETA(5)

TVRmax = THETA(6)

IC50=TVIC50

Imax=TVImax

Gama=TVGama

Rm=TVRm*EXP(ETA(1))
```

```
tp=TVtp      ; duration to achieve CST peak, assuming starting at 8AM
Kout=TVKout
Ra=TVRa
Rmax = TVRmax
TVCLhF = 297000      ; CL female (mL/h) at 500 kg
TVCLr = 3430
TVBmax1 = 121
TVKd = 0.387
TVKp2 = 1.04
TVKfs_b = 22.8      ; s=DEX_ISO    b=blood
TVKfp_b = 16.7      ; p=DEX_PHO
TVKfp_j = 30          ; j=joint
TVFs_b = 0.812
TVFp_b = 1
TVFd_j = 1        ; d=DEX
TVKas_m = 0.0115
TVKal_m = 0.131      ; l=DEX_ALC
TVKad_j = 4.94
TVExp_h = 0.75
TVExp_r = 0.75
TVCLhG = 242800        ;CL Male
Rb = 0.852
IF (SEX.EQ.0) THEN
    CLh = TVCLhF *EXP(ETA_CL) * (BW / 500) ** TVExp_h
ELSE
    CLh = TVCLhG *EXP(ETA_CL) * (BW / 500) ** TVExp_h
ENDIF
CLr = TVCLr * (BW / 500) ** TVExp_r
Bmax1 = TVBmax1
Kd = TVKd
Kp2 = TVKp2
```

```
If (DOSE.EQ.1) Then    ;    AMT CMT= 2
Kf = TVKfs_b
F_1 = TVFs_b
Ka = 0
End If
If (DOSE.EQ.2) Then ; AMT/Vp/Rb CMT=3
Kf = 0
F_1 = 0
Ka = 0
End If
If (DOSE.EQ.3) Then      ; AMT CMT=1
Kf = TVKfs_b
F_1 = TVFs_b
Ka = TVKas_m
End If
If (DOSE.EQ.4) Then        ; AMT CMT=2
Kf = TVKal_m
F_1 = 1
Ka = 0
End If
If (DOSE.EQ.5) Then        ; AMT CMT=2
Kf = TVKfp_b
F_1 = TVFp_b
Ka = 0
End If
If (DOSE.EQ.6) Then          ; AMT CMT=1
Kf = TVKfp_j
F_1 = TVFd_j
Ka = TVKad_j
End If
MWd = 392.46
```

```
If (PRODRUG.EQ. 0) MWo = 392.46        ; ALC

If (PRODRUG.EQ. 1) MWo = 516.41        ; DEX_PHO

If (PRODRUG.EQ. 2) MWo = 492.54        ; DEX_ISO

Vp = 70.72 * BW          ; mL

V1 = 13.045 * BW

V2 = 916.28*BW

Qco = 3570 * BW          ; mL/h

Q1 = 1184.63*BW

Q2 = 2395.44 * BW

CLu = 1.24 * BW              ; mL/h    (1.24 mL/h/kg)

Vbc = 4000                   ; mL, bladder capacity - trigger micturition

PI = 3.1415926

Tau=0.34

A_0(1) = 0

A_0(2) = 0

A_0(3) = 0

A_0(4) = 0

A_0(5) = 0

A_0(6) = 0

A_0(7) = Rm + Ra * COS(2 * PI / 24 * (-(tp-CLOCK_CCT+8)))

$DES

Kp1 = (-(A(4) - Kd - Bmax1) + ((A(4) - Kd - Bmax1) ** 2 + 4 * Kd * A(4)) ** 0.5) / (2 * Kd)        ;
nonlinear binding_liver

Ks = Kout*Rm + Kout*Ra*COS(2*PI/24*(TIME-(tp-CLOCK_CCT+8)))-
2*PI/24*Ra*SIN(2*PI/24*(TIME-(tp-CLOCK_CCT+8)))

If (STUDY_ID.EQ.5) Then

DADT(1) = -Kf * A(1)          ; Prodrug Amount_Depot

DADT(2) = Kf * A(1) * MWd / MWo - Ka * A(2)          ; DEX Amount_Depot

DADT(3) = (Ka * F_1 * A(2) + Q1 * A(4) / Kp1 + Q2 * A(5) / Kp2 - Qco * A(3) - CLr * A(3)) / Vp /
Rb    ; DEX conc_blood

Else

DADT(1) = -Ka * A(1)          ; Prodrug Amount_Depot
```

```
DADT(2) = Ka * A(1) - Kf * A(2)          ; Prodrug Amount_blood

DADT(3) = (Kf * F_1 * A(2) * MWd / MWo + Q1 * A(4) / Kp1 + Q2 * A(5) / Kp2 - Qco * A(3) - CLr * A(3)) / Vp / Rb    ; DEX conc_blood

End If

DADT(4) = (Q1 * (A(3) - A(4) / Kp1)) / V1 - (CLh * A(4) / Kp1) / V1        ; DEX conc_liver

DADT(5) = (Q2 * (A(3) - A(5) / Kp2)) / V2        ; DEX conc_remainder

DADT(6) = (CLr * A(3) - CLu * A(6)) / Vbc        ; DEX conc_urine

If (STUDY_ID .EQ. 1 .AND. TAD .LT. Tau) THEN

DADT(7) = Rmax/Tau + Ks * (1 - A(3) ** Gama * Imax / (A(3) ** Gama + IC50 ** Gama)) - Kout * A(7)          ; cortisol plasma

ELSE

DADT(7) = Ks * (1 - A(3) ** Gama * Imax / (A(3) ** Gama + IC50 ** Gama)) - Kout * A(7)

ENDIF

$ERROR

IPRED = F

Y = IPRED + ERR(1)

IRES = DV - IPRED

$THETA

(0.005, 0.038, 0.4) ;[IC50] 1

(18.6)    ;[Ra]2

(10,93,120) ;[Rm1] 3

(0.1, 0.933, 4) ;[tp] 4

(0.05, 0.25, 0.6) ;[Kout] 5

(1, 24, 60) ;[Rmax] 6

(10,47,90) ;[Rm2] 7

(0.7,1,1.3) ;[gama] 8

$OMEGA

  0.15 ;[Rm]

$SIGMA

  403 ; Additive

$PRIOR NWPRI
```

```
$THETAP
(0.0383 FIX)      ;[IC50]
(18.6 FIX)    ;[Ra]
(93 FIX)    ;[Rm1]
(0.933 FIX) ;[tp]
(0.25 FIX) ;[Kout]
(24.8 FIX)    ;[Rmax]
(47 FIX)    ;[Rm2]
(1 FIX) ;[gama]
$THETAPV BLOCK(8)
  0.0000116 ;IC50
  0.00 1.78 ; Ra
  0.00 0.00 0.22    ; Rm1
  0.00 0.00 0.00 0.0324 ; tp
  0.00 0.00 0.00 0.00 0.000392    ; Kout
  0.00 0.00 0.00 0.00 0.00 2.23    ; Rmax
  0.00 0.00 0.00 0.00 0.00 0.00 0.69    ; Rmax
  0.00 0.00 0.00 0.00 0.00 0.00 0.00 0.01    ; gama
$OMEGAP BLOCK(1)
  (0.15)    ;[Rm]
$OMEGAPD    (4.0 FIX)
$SIGMAP
  (403) ;[add]
$SIGMAPD    (1.0 FIX)
$EST METHOD=COND INTERACTION MAXEVAL=999 NSIG=6 PRINT=5 NOABORT SIGL=10
MCETA=10                            ; FOCEI+Priors
$COV MATRIX=S PRINT=E UNCONDITIONAL
$TABLE ID TIME DV PHASE_ID TAD AMT RATE CMT DATATYPE MDV EVID BW AGE SEX
BREED STUDY_ID ROUTE SEASON DATAQUALITY IPRED PRED ONEHEADER NOPRINT
FORMAT=s1PE13.7 FILE=mytabC8
$TABLE ID TIME DV PHASE_ID TAD CMT DATATYPE MDV PRED IPRED CWRESI CWRES
ONEHEADER NOPRINT FORMAT=s1PE13.7 FILE=sdtabC8
$TABLE ID TIME TAD DV IPRED CMT BW AGE SEX BREED ROUTE CONDITION SEASON
```

IC50 Gama Rm Ra Rmax tp Kout ETA1 ONEHEADER NOPRINT NOAPPEND FORMAT=s1PE13.7 FILE=patabC8

References

1. Yu R, &Jusko WJ (2025) Meta-Analysis and Mechanism-Based Modeling of Synovial and Plasma Pharmacokinetics and Adrenal Suppression Following Intra-Articular Injection of Methylprednisolone Acetate in Horses. *J Vet Pharmacol Ther*. DOI:10.1111/jvp.13504

2. Toribio RE (2007) Essentials of equine renal and urinary tract physiology. *Vet Clin North Am Equine Pract* 23(3):533-61, v. DOI:10.1016/j.cveq.2007.09.006
